# Hard Selective Sweep and Ectopic Gene Conversion in a Gene Cluster Affording Environmental Adaptation

**DOI:** 10.1371/journal.pgen.1003707

**Published:** 2013-08-22

**Authors:** Marc Hanikenne, Juergen Kroymann, Aleksandra Trampczynska, María Bernal, Patrick Motte, Stephan Clemens, Ute Krämer

**Affiliations:** 1 Functional Genomics and Plant Molecular Imaging, Center for Protein Engineering (CIP), Department of Life Sciences, University of Liège, Liège, Belgium; 2 Laboratoire d'Ecologie, Systématique et Evolution, Université Paris-Sud/CNRS, Orsay, France; 3 Department of Plant Physiology, University of Bayreuth, Bayreuth, Germany; 4 Department of Plant Physiology, Ruhr University Bochum, Bochum, Germany; Harvard University, United States of America

## Abstract

Among the rare colonizers of heavy-metal rich toxic soils, *Arabidopsis halleri* is a compelling model extremophile, physiologically distinct from its sister species *A. lyrata*, and *A. thaliana*. Naturally selected metal hypertolerance and extraordinarily high leaf metal accumulation in *A. halleri* both require *Heavy Metal ATPase4* (*HMA4*) encoding a P_IB_-type ATPase that pumps Zn^2+^ and Cd^2+^ out of specific cell types. Strongly enhanced *HMA4* expression results from a combination of gene copy number expansion and *cis*-regulatory modifications, when compared to *A. thaliana*. These findings were based on a single accession of *A. halleri*. Few studies have addressed nucleotide sequence polymorphism at loci known to govern adaptations. We thus sequenced 13 DNA segments across the *HMA4* genomic region of multiple *A. halleri* individuals from diverse habitats. Compared to control loci flanking the three tandem *HMA4* gene copies, a gradual depletion of nucleotide sequence diversity and an excess of low-frequency polymorphisms are hallmarks of positive selection in *HMA4* promoter regions, culminating at *HMA4-3*. The accompanying hard selective sweep is segmentally eclipsed as a consequence of recurrent ectopic gene conversion among *HMA4* protein-coding sequences, resulting in their concerted evolution. Thus, *HMA4* coding sequences exhibit a network-like genealogy and locally enhanced nucleotide sequence diversity within each copy, accompanied by lowered sequence divergence between paralogs in any given individual. Quantitative PCR corroborated that, across *A. halleri*, three genomic *HMA4* copies generate overall 20- to 130-fold higher transcript levels than in *A. thaliana*. Together, our observations constitute an unexpectedly complex profile of polymorphism resulting from natural selection for increased gene product dosage. We propose that these findings are paradigmatic of a category of multi-copy genes from a broad range of organisms. Our results emphasize that enhanced gene product dosage, in addition to neo- and sub-functionalization, can account for the genomic maintenance of gene duplicates underlying environmental adaptation.

## Author Summary

Existing genetic diversity reflects evolutionary history, but it has rarely been possible to probe for footprints of selection at loci known to functionally govern adaptive traits. Both naturally selected metal hypertolerance and extraordinary leaf metal accumulation of the extremophile *Arabidopsis halleri* require strongly enhanced transcript levels of *Heavy Metal ATPase4* (*HMA4*) encoding a P_IB_-type ATPase that pumps Zn^2+^ and Cd^2+^ out of specific cells. By comparison to the metal-sensitive *A. thaliana*, highly elevated *HMA4* expression results from a combination of gene copy number expansion and *cis*-regulatory modifications. But how do these findings, which were based on a single accession, relate to species-wide *HMA4* sequence diversity in *A. halleri*? Addressing this question, we detect positive selection in the promoter regions of three tandem *A. halleri HMA4* paralogs, which are uniformly *cis*-activated. The accompanying hard selective sweep, however, is segmentally eclipsed as a consequence of recurrent ectopic gene conversion among *HMA4* protein-coding sequences, which undergo concerted evolution. Together, this constitutes an unexpectedly complex profile of polymorphism as a result of natural selection. Our observations can serve as a blueprint for future analyses of duplicated genes that have undergone selection for more of the same gene product.

## Introduction

Analyses of nucleotide sequence variation bear great promise for advancing our understanding of evolutionary processes. However, such analyses have so far rarely targeted loci of experimentally established roles in naturally selected adaptive traits, and, instead, have mostly been conducted on candidate loci or even anonymous sequences [1]–[3]. Among the highest selection pressures known in ecology are those encountered by plants on metalliferous soils, which contain high, toxic levels of heavy metals from geological anomalies or anthropogenic contamination [4]. Examples of metalliferous soils are the widespread ultramafic (serpentine) soils rich in Ni, Co and Cr, and calamine soils containing high levels of Zn, Cd, and Pb. The extremophile species *Arabidopsis halleri* is one of the few plant taxa capable of colonizing calamine metalliferous soils [5]. In addition to its hypertolerance to Zn, Cd and likely Pb, *A. halleri* groups among approximately 500 known taxa of so-called hyperaccumulators of metals such as Ni, Co, Zn or Cd [6], [7]. Hyperaccumulators are characterized by leaf metal concentrations exceeding those of ordinary non-accumulator plants by more than two orders of magnitude. Metal hyperaccumulation contributes to metal hypertolerance and has been proposed to act as an elemental defense against biotic stress [8], [9].

*A. halleri* is closely related to *Arabidopsis lyrata* and to the genetic model plant *Arabidopsis thaliana*, both of which are non-hyperaccumulators and exhibit only basal metal tolerance common to all vascular plants [10]. Different from *A. thaliana*, *A. halleri* is an outcrossing, stoloniferous perennial, with a nuclear genome of 2 *n* = 16 chromosomes [6]. In an attempt to address the molecular basis of Zn and Cd hyperaccumulation and associated hypertolerance in *A. halleri*, cross-species transcriptomics approaches employing the accession Langelsheim (Germany) established dozens of candidate genes with potential functions in metal homeostasis, of which transcript levels were elevated in *A. halleri* when compared to *A. thaliana* [11]–[13]. Functional characterization through various molecular approaches supported a role for several of these genes including *HEAVY METAL ATPASE4* (*HMA4*) [8], [12], *HMA3* [11], *METAL TRANSPORT PROTEIN1* (*MTP1*) [11], [14], *NICOTIANAMINE SYNTHASE2* (*NAS2*) [13], [15], and *IRON-REGULATED TRANSPORTER3* (*IRT3*) [6], [7], [16]. Transcript abundance of *HMA4* was highest of all identified candidate genes, with more than 100-fold higher transcript levels in both roots and shoots of *A. halleri* than in *A. thaliana* or *A. lyrata* [12], [17]. The HMA4 protein is a plasma membrane transport protein acting in ATP-driven cellular export-mediated detoxification of Zn^2+^ and Cd^2+^, as well as root-to-shoot translocation of both metals [8], [18]. The strongly enhanced *HMA4* transcript levels present in *A. halleri* were shown to be necessary not only for metal hypertolerance but also for metal hyperaccumulation, by employing RNA interference-mediated silencing in the *A. halleri* accession Langelsheim. The introduction into *A. thaliana* of an *AhHMA4* promoter fused to an *AhHMA4* cDNA suggested that *AhHMA4* alone, however, is not sufficient to generate either metal hypertolerance or hyperaccumulation [8]. In agreement with these findings, genetic studies identified *HMA4* and *MTP1* to be located within rather large QTL regions for metal hypertolerance in a segregating back-cross 1 population of an inter-specific hybrid cross between *A. halleri* (accession Auby, France) and *A. lyrata* [17], [19]. Moreover, *HMA4* co-localized with one out of several major QTL for leaf Zn and Cd hyperaccumulation, respectively, in a segregating F_2_ population [20], [21]. Among the candidate genes of *A. halleri* characterized in detail to date, *HMA4* thus makes the largest contribution to both metal hyperaccumulation and metal hypertolerance.

High *HMA4* transcript levels were shown to be attributable to a combination of tandem gene triplication and *cis*-activation in the Langelsheim accession of *A. halleri* [8]. Promoter-reporter fusions suggested approximately equivalent quantities and localizations of promoter activity for all three *A. halleri HMA4* gene copies, in agreement with copy-specific transcript quantification through quantitative real-time RT-PCR [8]. Because of almost identical protein-coding sequences, the functions of the three HMA4 protein isoforms of *A. halleri* have not been individually characterized. All these findings supported a critical role of enhanced *HMA4* gene product dosage in naturally selected metal hyperaccumulation and hypertolerance of *A. halleri* [8]. Interestingly, high *HMA4* transcript levels, copy number expansion and *cis*-activation were also reported in *Noccaea caerulescens* [22], [23], another Zn/Cd hyperaccumulator in the Brassicaceae family, in which metal hyperaccumulation and associated hypertolerance must have evolved independently. Moreover, copy number expansion appears to be common among additional highly expressed metal hyperaccumulation/hypertolerance candidate genes of *A. halleri*, for example the *ZINC-REGULATED TRANSPORTER, IRON-REGULATED TRANSPORTER-RELATED PROTEIN* (*ZIP*) genes *ZIP3*, *ZIP6* and *ZIP9* [12], *MTP1* [14], [24] and *PLANT DEFENSIN* (*PDF*) genes [25].

Gene duplication is known as a major driver of genome evolution over long timescales [26]. In eukaryotic genomes, gene duplications occur spontaneously at rates that are between 100 and 10,000 times higher per locus than those of base substitutions per site [27], [28], thus explaining the presence of substantial gene copy number variation polymorphism in genomes. For example, per haploid genome and generation, *S. cerevisiae* was estimated to spontaneously acquire about 0.002 non-synonymous base substitutions within coding regions and 0.02 gene duplications [28]. A number of genetic diseases of humans are caused by gene duplication events [29], [30]. Current theory predicts the rapid loss of recent duplicates unless they undergo neo- or sub-functionalization, with few exceptions [26], [31], [32]. However, the factual contribution of gene duplication to evolutionary adaptation as an outcome of natural selection remains poorly understood. Functional diversity and evolutionary dynamics of multigene families are of particular importance in plant and animal immunity, as exemplified by plant Resistance (*R*) and human Major Histocompatibility Complex (*MHC*) genes [33]. Natural selection for increased gene product dosage was implied to account for copy number expansion of the *BOT1* boron tolerance locus of barley [34], the *MATE1* aluminum tolerance locus of maize [35] and the human salivary amylase gene (*AMY1*) [36]. However, these reports were based merely on functional data encompassing genotype-phenotype relationships, without evidence for selection from an analysis of sequence polymorphism.

Here, we address two gaps in present knowledge, namely whether a signature of selection can indeed be identified at a locus known to functionally govern an adaptive trait and, more specifically, whether positive selection for increased gene product dosage can result in the fixation of gene duplications [37]. We detect positive selection at the copy-number expanded *HMA4* metal hypertolerance locus of *Arabidopsis halleri*. Moreover, we show that the profile of polymorphism is unexpectedly complex as a result of ectopic gene conversion. This work can act as a guide for related studies on other duplicated genes, and warrants caution in targeted analyses as well as genome-wide scans of polymorphism when dealing with presently or historically copy-number expanded loci.

## Results/Discussion

### Evidence for Positive Selection in *HMA4* Promoter Regions

**Figure 1:**
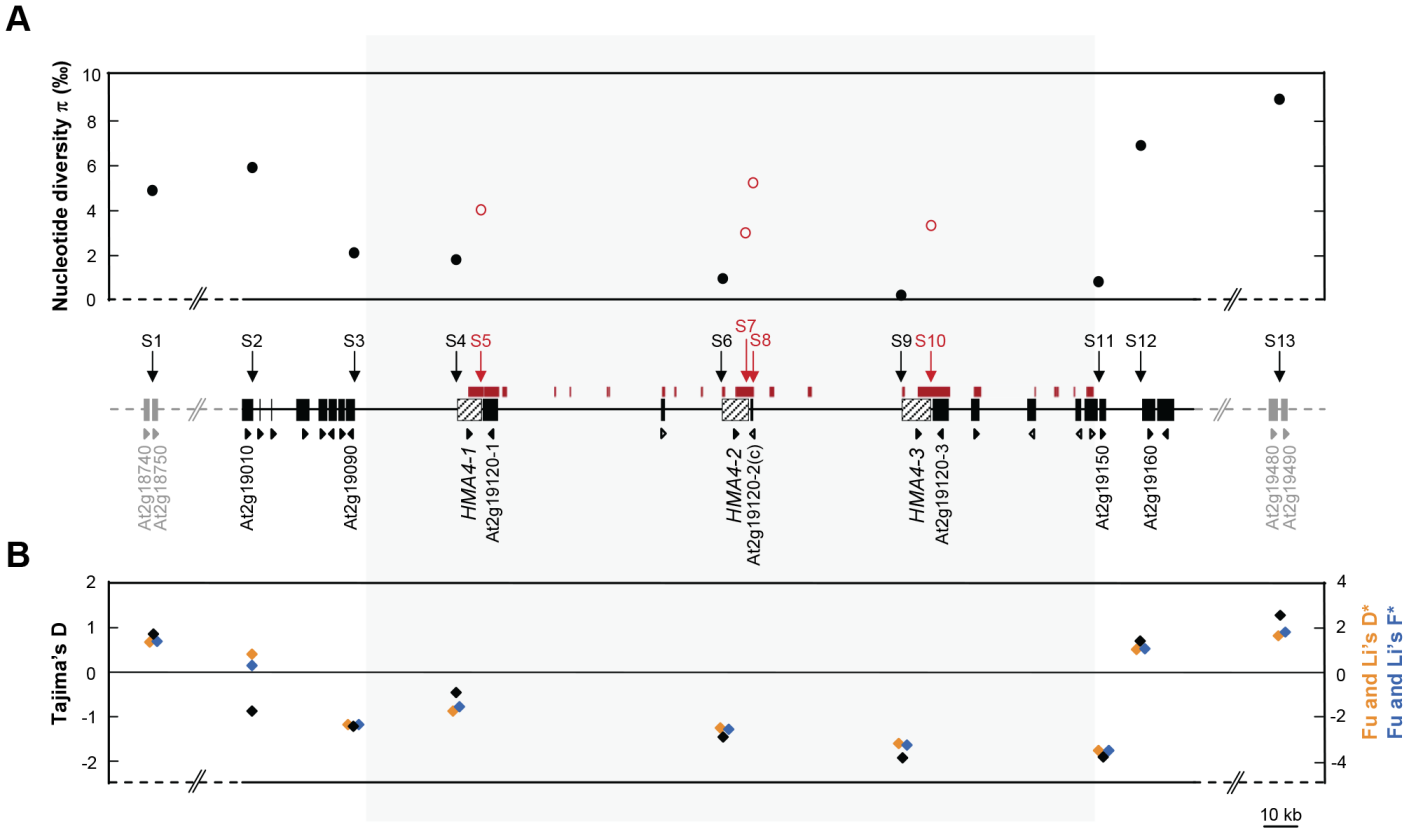
Nucleotide sequence diversity across the *HMA4* genomic region in *A. halleri*. (A) Average pairwise nucleotide sequence diversity π across the *HMA4* genomic region in *A. halleri* ssp. *halleri*. (B) Molecular population genetic statistical tests for the deviation from neutrality, Tajima's *D*, Fu and Li's D*, and Fu and Li's F* in *A. halleri* ssp. *halleri*. Differently colored symbols are defined by concordantly colored axis titles. Vertical arrows indicate positions of sequenced segments. The region of *HMA4* triplication is shaded in grey. Marked in red color are stretches of sequence that are present in several, almost identical copies (horizontal bars; detected using NCBI MEGABLAST program with default settings and a word size of 256; see Table S3), and sequenced segments therein (arrows, fonts, red open symbols instead of closed black symbols in panel (A)). Direction of transcription (triangular arrow) is given for each gene (rectangle, Arabidopsis Genome Identifier code of *A. thaliana* ortholog), with genes of unknown position in *A. halleri* shown in grey. For additional information see Table S2.

For an analysis of intra-specific nucleotide sequence diversity across the triple *HMA4* genes of *A. halleri*, we sequenced from multiple individuals (Table 1) series of 13 genomic DNA segments positioned consecutively along the 150-kb *HMA4* region and in flanking regions (Figure 1, Figure S1A and Table S1). In more detail, amplicons of between 492 and 2245 bp in length (see Table S1) were designed based on published sequence data, and sequenced from between 15 and 20 individuals (see Table S2; http://www.ebi.ac.uk, accession nos. HE995813 to HE996227). The number of alleles observed per genotype never exceeded expectations of a maximum of two for any of the amplicons (see Table S2, lower section; see Materials and Methods section ‘Sequencing, Sequence Assembly and Assignment of Consensi’). We confirmed leaf metal accumulation in these same individuals by Inductively-Coupled Atomic Emission Spectrometry analysis of field-collected leaves. Maximal concentrations exceeded 10,000 µg Zn g^−1^ leaf dry biomass and 100 µg Cd g^−1^ leaf dry biomass in individuals from both non-metalliferous and metalliferous sites that are characterized by toxic levels of metals in the soil and a specialist vegetation (Table 1). For comparison, we also obtained nucleotide sequence data from single individuals of the Zn/Cd-hypertolerant and -hyperaccumulating subspecies *A. halleri* ssp. *gemmifera* [38] from East Asia and the closely related Zn/Cd-sensitive, non-hyperaccumulating *Arabidopsis lyrata*. The genome of *A. lyrata* contains a single functional *HMA4* gene (Figure S1B) in a region that is overall syntenic to *A. halleri* (Figure 1 and S1A) and *A. thaliana* (Figure S1C) [39]. In addition, the *A. lyrata* genome uniquely contains a second, 5′-truncated *HMA4*-like pseudogene in a non-syntenic position.

**Table 1:** Origin of *Arabidopsis halleri* individuals and other plants used in this study.

Collection site		Individuals			GPS		Soil conc. (mg kg^−1^)		Leaf conc. (mg kg^−1^)	
#	Name	Type	*n*	Species	N	E	Zn	Cd	Zn	Cd
1	Langelsheim	M	3	*A. halleri* ssp. *halleri*	51 56.569	10 20.955	1,200–3,200	7.2–15	7,200–11,300	24–190
2	Oker	M	3	*A. halleri* ssp. *halleri*	51 53.750	10 29.009	n.a.		11,600	12
3	Eckertal	M	2	*A. halleri* ssp. *halleri*	51 52.690	10 38.298	1,000–1,200	0.6–0.7	14,800–17,000	24–38
4	Schierke Road	NM	1	*A. halleri* ssp. *halleri*	51 45.413	10 41.147	20	0.5	6,100	56
5	Schierke	M	3	*A. halleri* ssp. *halleri*	51 44.960	10 41.467	200–1,300	0.6–1.3	5,300–11,300	0.6–10
6	Rodacherbrunn	NM	3	*A. halleri* ssp. *halleri*	50 25.623	11 33.811	3.4–6.3	0.3–0.6	4,700–8,800	58–220
7	Stutenkamm	NM	3	*A. halleri* ssp. *halleri*	50 24.724	11 33.315	4.4–11	0.2–0.4	2,900–6,600	15–100
8	Auby, FR^a^	M	1	*A. halleri* ssp. *halleri*			n.a.		n.a.	
9	Tada Mine, JP^b^	M	1	*A. halleri* ssp. *gemmifera*	34 53.728	135 21.044	n.a.		n.a.	
-	MN47^c^	NM	1	*A. lyrata* ssp. *lyrata*	-	-	n.a.		n.a.	

Countries of sampling outside Germany are specified. Extractable soil and total leaf concentrations are shown relative to dry mass. M, metalliferous site; NM, non-metalliferous site. Sources of genotypes:

a BC1 parent [17], [72];

b Fujita Co., Japan;

c Nottingham Arabidopsis Stock Centre.

n.a.: not analyzed.

If a novel mutation confers a strong selective advantage, the corresponding haplotype is likely to sweep through a population. This reduces or even eliminates pre-existing nucleotide sequence diversity at the affected locus and – proportionately to the extent of genetic linkage – at flanking loci through genetic hitchhiking [40]. In order to test for evidence of a selective sweep in the *HMA4* genomic region of *A. halleri*, we calculated statistics of genetic diversity. At distant control loci (S1 and S13) and loci flanking the ∼150-kb *HMA4* genomic region (S2 and S12), average pairwise nucleotide sequence diversity (π) was between 4.9 and 9.1‰ (Figure 1A and Table S2), and thus within the published range for random neutral loci in *A. halleri* [41]–[43]. Comparable studies on *A. halleri* ssp. *halleri* have reported a median π of 3.9‰ (between 0.3 and 37.7‰) for 24 randomly chosen loci [42] and a median π of 4.3‰ (between 1.8 and 32.7‰) for a total of 8 loci [41] (Figure S2). Indeed, this was in sharp contrast with much lower values for π of between 0.1 and 1.8‰ for segments comprising sequences in the promoter regions of the three paralogous *HMA4* gene copies (S4, S6, S9; Figure 1A, Figure S1D and Table S2). Compared to the distant and flanking control loci, π decreased gradually towards and within the *HMA4* region and reached a minimum of 0.1‰ at the *HMA4-3* promoter (S9), yielding a profile as expected upon a hard selective sweep. This characteristic profile of nucleotide sequence diversity was found to be interrupted, however, by elevated π values of between 3.2 and 5.2‰ for segments positioned within the coding sequences of the three *HMA4* gene copies (S5, S7, S10) and also for the additional segment S8, all comprising sequences that are present in two or more, almost identical copies in the *HMA4* genomic region (Figure 1A, Tables S2 to S4). The overall profile of nucleotide sequence diversity across the *HMA4* region was robust against error in sequence assignment to S5, S7 and S10 (see Materials and Methods, Table S4A), as well as towards a regionally separate analysis of individuals from the Harz Mountains and the Thuringian Forest (Table S4B).

**Figure 2:**
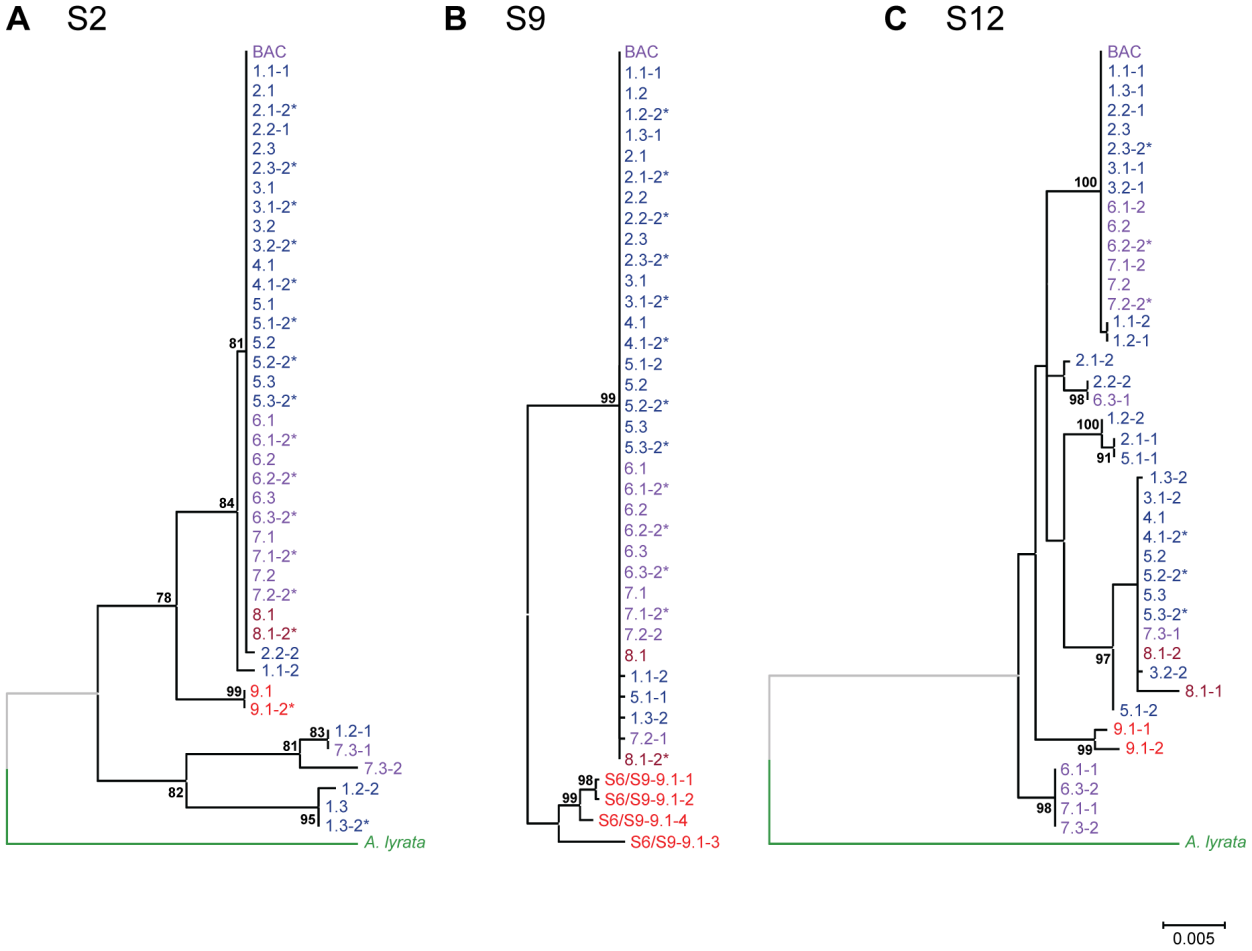
Phylogenetic trees for segments comprising unique sequences in the genomic *HMA4* region of *A. halleri*. Shown are maximum likelihood trees for amplicons (A) S2, (B) S9, and (C) S12. Alleles are named according to *A. halleri* individuals ([collection site].[individual], see Table 1) and are color-coded based on the population of their origin: blue, Harz Mountains (1 to 5); violet, Thuringian Forest (6 and 7, BAC); brown, Auby (8.1); red, *A. halleri* ssp. *gemmifera* (9.1). Published sequences from *A. halleri* BACs were included (Genbank accession numbers EU382073.1 and EU382072.1) [8], and *A. lyrata* ssp. *lyrata* sequences [39] are shown as outgroup (green) where possible. Percentages of bootstrap support (1000 replicates) of a minimum of 75% are given at the corresponding nodes. Branch lengths are scaled by the number of substitutions per site. The datasets were as follows (number of sequences × number of aligned positions in bp): S2: 42×1464 (A); S9: 39×3710 (B); S12: 42×2480 (C). Asterisks (*) denote the second alleles that were inferred in individuals from which only a single sequence was obtained and which were thus concluded to be homozygous for the respective locus. Note that in *A. halleri* ssp. *gemmifera* (individual 9.1), primer pairs designed to obtain S6 and S9 (see Table S1) both yielded the same set of four highly similar sequences (see Figure S1D).

To further substantiate the evidence for positive selection in the genomic *HMA4* region of *A. halleri*, we conducted statistical tests of molecular population genetics by calculating Tajima's *D*, Fu and Li's D*, and Fu and Li's F* [44], [45]. For segments in the promoter regions of *HMA4-1* (S4), *HMA4-2* (S6) and *HMA4-3* (S9), these three tests unanimously indicated an excess of rare polymorphisms resulting from a depletion of higher-frequency, ancestral polymorphisms. A statistically significant deviation from expectations under neutral evolution was detected at the promoter of *HMA4-3* (S9; Figure 1B and Table S2), diagnostic of positive selection. Indeed, diversity statistics indicated a unique combination of a very low value for π with a highly negative Tajima's D for S9 (see Figure S2). In agreement with these results, there were fewer long and intermediate-length branches in the topologies of maximum likelihood phylogenetic trees for *HMA4* (S4, S6, S9) than observed for control loci on either side of the *HMA4* region (S1, S2, S12, S13; Figure 2, Figure S3) [44]. In the region of extremely low sequence diversity in the promoter region of *HMA4-3* (S9) of *A. halleri* ssp. *halleri* (see Figure 1A), for example, all polymorphisms were unique to single observations (e.g., Figure 2B, 1.1-2, 1.3-2, 5.1-1, 7.2-1; see also Figure 1B). Taken together, statistical tests of sequence diversity, molecular population genetics and sequence phylogenies concordantly support a hard selective sweep centered on the promoter of *HMA4-3*, with genetic hitchhiking [40] covering a total of 250 kb. This is comparable to previously reported selective sweeps, which affect chromosomal regions of between 60 and 600 kb in length linked to domestication loci of crop plants [46].

### *HMA4* Transcript Levels

**Figure 3:**
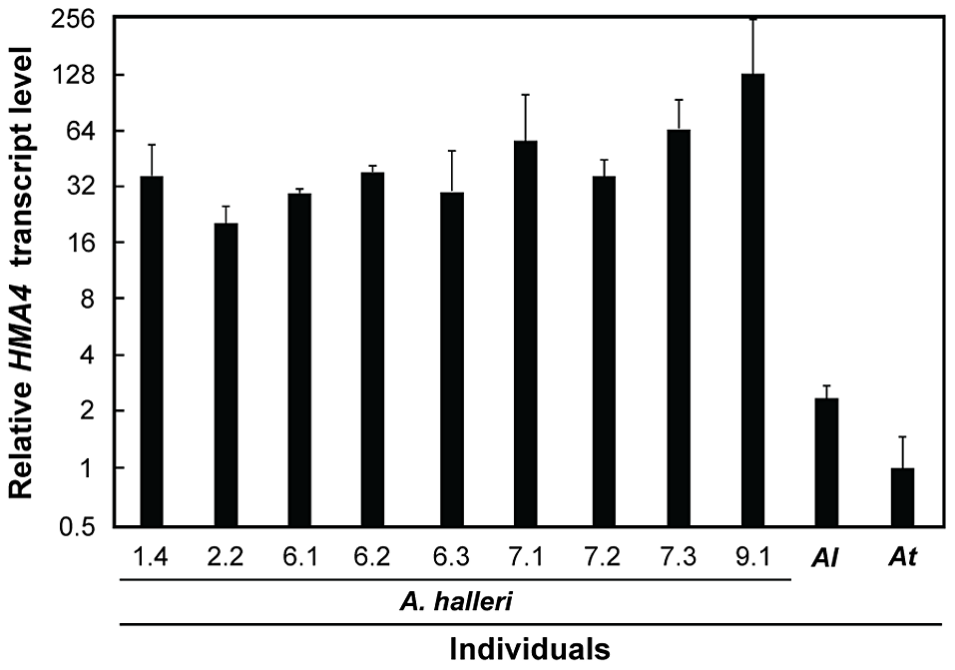
Leaf *HMA4* transcript levels in *A. halleri* individuals. Values (arithmetic means ± s.e.m. of two independent biological experiments) are given relative to *HMA4* transcript levels in *A. thaliana* (*At*). Nucleic acids were extracted from leaves of vegetative plants, including *A. lyrata* (*Al*), grown on soil in a greenhouse.

As demonstrated in a single individual of *A. halleri* [8], the combination of gene copy number expansion and *cis*-regulatory divergence results in strongly enhanced steady-state *HMA4* transcript levels that are necessary for metal hyperaccumulation and hypertolerance. If this was selected for in the entire species *A. halleri*, as indicated by the diversity statistics (see Figure 1), then we would expect high *HMA4* transcript levels in all *A. halleri* individuals. Indeed, we observed between 20- and 130-fold higher *HMA4* transcript levels across individuals of *A. halleri* from different collection sites, when compared to *A. thaliana* (Figure 3). This result supports a substantial increase in *HMA4* gene product dosage in all *A. halleri* ssp. *halleri* and ssp. *gemmifera* individuals analyzed here, by comparison to *A. lyrata* and *A. thaliana*.

### Ectopic Gene Conversion among Protein-Coding Sequences of *HMA4* Paralogs

**Figure 4:**
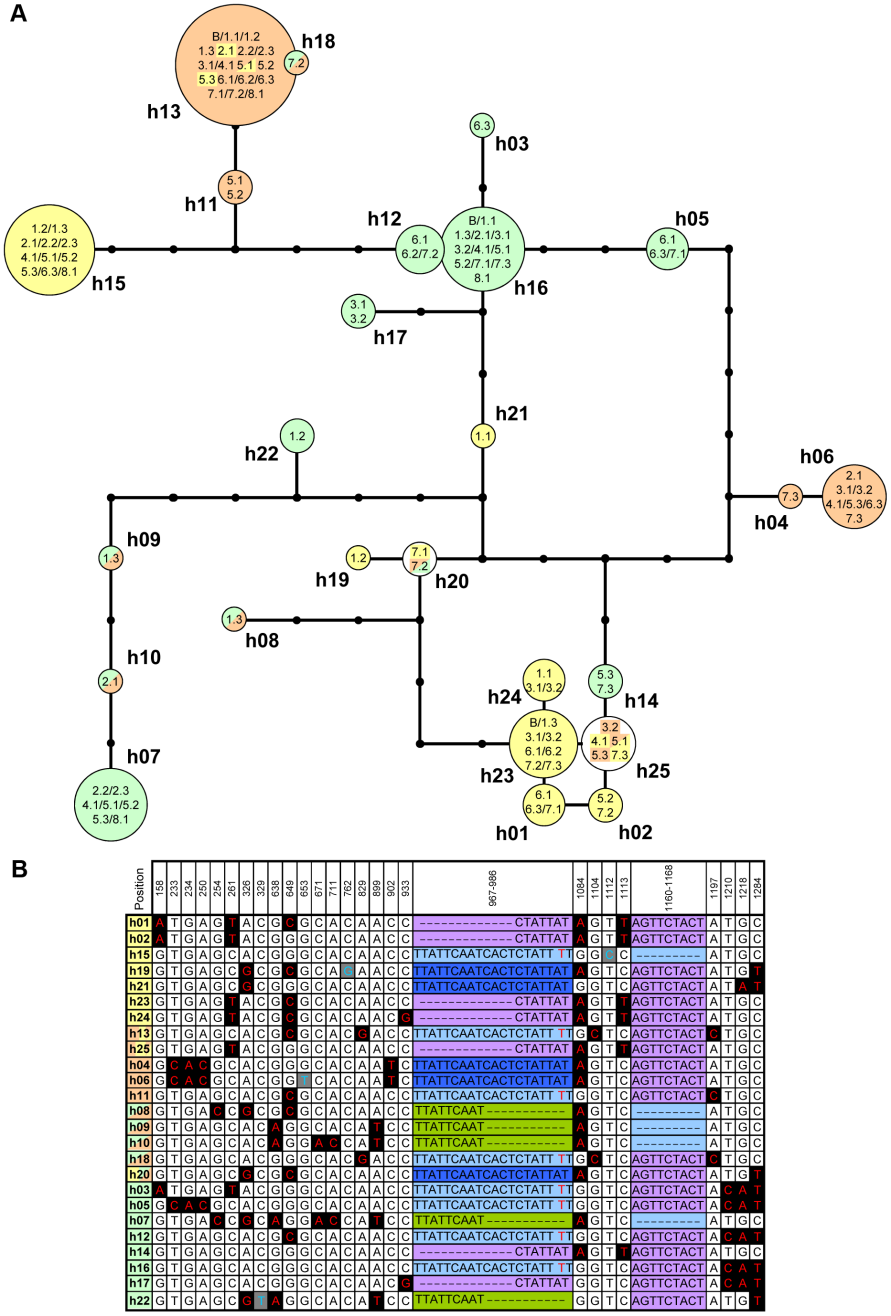
Recurrent sequence exchange between coding regions of *A.* **halleri HMA4**** **gene copies.** **** (A) Network analysis of consensus sequences of 3′ *HMA4* coding regions of *A. halleri* ssp. *halleri*. Each node represents one mutational step. Node size corresponds to the number of alleles, as specified inside nodes ([collection site].[individual], see Table 1), constituting the respective consensus (h01 to h25). Alleles were assigned to *HMA4*-*1* (green, S5), -*2* (yellow, S7) or -*3* (orange, S10, see Figure 1), with multiple coloration for both ambiguous assignments and assignments to multiple gene copies. Note that assignment to *HMA4-2* was based directly on sequence data, whereas for all sequences not derived from BAC (B) data, assignment to *HMA4-1* or *HMA4-3* was inferred (Materials and Methods). (B) Polymorphic positions in consensus sequences. Header row refers to the alignment of consensi of segments S5/S7/S10 from *A. halleri*. SNPs present in single (blue fonts) or multiple (red fonts) consensi are highlighted. For indels, each color marks one allele.

For segments located within coding sequences of the three *HMA4* gene copies (S5, S7, S10), relationships among haplotypes differed from those for segments located in *HMA4* promoters (S4, S6, S9). Phylogenetic reconstructions of S5, S7 and S10 did not recover three distinct groups of haplotypes as expected for three independently evolving paralogs (Figure S4). Instead, the genealogy resembled a network-like structure, with complex relationships between *HMA4* haplotypes at different loci (Figure 4A). For example, out of a total of 25 haplotypes, three were found at two or more of the paralogous *HMA4* genes (h13, h20, h25; Figure 4B). These results demonstrate a recurrent transfer of genetic information between the coding sequences of different *HMA4* gene copies of *A. halleri*.

**Figure 5:**
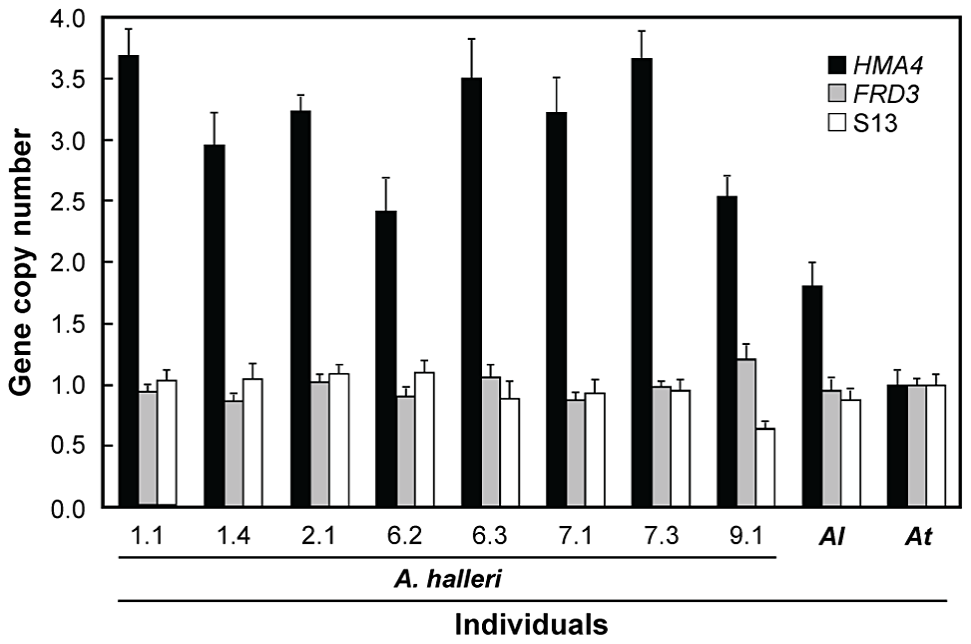
*HMA4* gene copy number in field-collected individuals of *A.* **halleri**** **.** **** Values (arithmetic means ± s.e.m., *n* = 16 technical replicates) are given relative to single-copy control loci *FRD3* [12] and S13 (see Figure 1). *A. thaliana* (*At*) genomic DNA served as calibrator. Note that one of the two *HMA4* gene copies detected in *A. lyrata* (*Al*) corresponds to a truncated pseudogene (see Figure S1B).

Segmental transfer of genetic information between paralogous sequences can arise in somatic cells during homologous recombination-based repair of double-strand breaks, addressed here as ectopic gene conversion (EGC, also termed interlocus or non-allelic gene conversion), or alternatively result from unequal crossing-over events during meiosis [29], [47], [48]. Quantitative PCR analysis of genomic DNA of *A. halleri* individuals from different collection sites was consistent with the species-wide presence of three *HMA4* gene copies per haploid genome (Figure 5). Average gene copy number was estimated at 3.2±0.2 for *A. halleri*, compared to 1.8±0.2 and 1.0±0.1 for *A. lyrata* and *A. thaliana*, respectively (arithmetic means ± SD), whereby one of the two gene copies detected in *A. lyrata* is a truncated pseudogene in a non-syntenic position (see Figure S1B). A total of three *HMA4* gene copies is in agreement with our observations of a maximum of six alleles observed per individual upon joint PCR amplification of all 3′ *HMA4* coding sequences (S5/S7/S10; Table S2), and a maximum of two alleles observed in the promoter region of each *HMA4* gene copy (S4, S6, S9; Table S2). The lack of evidence for *HMA4* copy number variation among *A. halleri* individuals suggests that recurrent EGC events account for the segmental transfer of genetic information between paralogous *HMA4* coding regions. EGC is known to be common among some genes, for example rRNA genes [29], [47], [49]–[51]. Paralogous genes of eukaryotes have been reported to exchange sequence information at per-locus frequencies even higher than those of spontaneous gene duplications [52], [53], thus contributing significantly to human disease [54]. The contribution of EGC to adaptation, however, is poorly understood.

EGC is predicted to transfer a newly arisen mutation from the site of its origin in one *HMA4* paralog to the corresponding sites in the other two paralogs, thus cumulatively enriching species-wide sequence diversity in each individual *HMA4* gene copy [55]. This explains the higher levels of nucleotide sequence diversity detected at S5, S7, S8 and S10, when compared to S4, S6 and S9 (see Figure 1) [29], [32]. Simultaneously, EGC suppresses between-copy sequence divergence and thus results in the concerted evolution of the affected loci [29]. Our findings imply that EGC accounts for the high extent of 99 to 99.3% inter-copy sequence identity among *A. halleri HMA4-1* to *-3* coding sequences (Table S3) [8], consistent with the prevalence of EGC among duplicates of >95% sequence identity known in other organisms [29], [47].

Hallmarks of EGC were also detected in the multi-copy portion of segment S8 outside the *HMA4* coding sequence (Figure S5A, Table S3), again with a network-like genealogy (Figure S5B–D) and a comparably high π of 9.5‰ (as opposed to π of 1.7‰ for the single-copy 3′-portion of S8). As in *A. halleri* ssp. *halleri*, EGC was also evident among the coding sequences of *HMA4* gene copies of *A. halleri* ssp. *gemmifera* (Figure S5D and S6), with an apparent additional EGC event between the promoters of *HMA4-2* and *-3* that was uniquely observed in this individual (S6, S9; see Figure S1D and compare Figure 2B and Figure S3C and S3D).

Population genetics theory and simulations have been developed for small multigene families undergoing concerted evolution [56]–[59]. Nucleotide substitution rates were predicted to be strongly enhanced with increasing gene copy number for beneficial mutations, whereas gene copy number had no effect on substitution rates for selectively neutral mutations [57]. Indeed, the *AhHMA4* protein-coding sequences represented in S5/S7/S10, which correspond to the cytoplasmic C-terminal regulatory domain of the HMA4 protein [60], show an over-proportionately high nucleotide sequence divergence of 22% from *A. thaliana* [8]. By comparison, within coding regions in general, average divergence of both *A. halleri* and *A. lyrata* from *A. thaliana* is around 6%. In the corresponding region of *HMA4*, *A. lyrata* is 9% divergent from *A. thaliana* and 22% divergent from *A. halleri*. This suggests an enhanced rate of fixed sequence alterations in 3′ *AhHMA4* coding sequence of S5/S7/S10, which – according to theoretical considerations – is likely to constitute evidence for positive selection [57]. Different from predictions, however, there is no prevalence of non-synonymous over synonymous nucleotide substitutions in this region, but a prevalence of indel polymorphisms instead. Nonetheless, these considerations suggest that *HMA4* gene copy number expansion is not only a result of selection for enhanced gene product dosage, but – in combination with EGC – accommodates an enhanced evolutionary rate of *HMA4* under positive directional selection.

Regions of the human genome hosting multigene families that undergo segmental exchange of sequence information have been addressed as hypermutable [8], [30]. Similarly, sequence exchange was proposed to contribute to the unusually high levels of sequence diversity among plant disease Resistance (*R*) genes, which typically belong to multigene families and are often present in the genome as tandem arrays of multiple paralogous genes [61]. Alongside unequal crossing over and illegitimate inter-allelic recombination, EGC was implicated in the generation of novel pathogen recognition specificities [33], [61]–[64]. The pervasiveness of sequence differences between paralogous *R* genes, despite sequence exchange, was attributed to small exchanged tracts of sequence of mostly <100 bp among multiple paralogs [61], [63], [64], to the suppression of unequal crossing over within *R* gene clusters of homozygotes [61], to the occurrence of inter-allelic rather than inter-locus gene conversion [33], or to the past discontinuation of sequence exchange [65]. By comparison to the high sequence diversification among paralogous *R* genes, the concerted evolution of *A. halleri HMA4* paralogs is in stark contrast. This could be interpreted to indicate a prominent role for selection in determining the outcome of inter-locus sequence exchange, a process that appears to be common at least in some classes of multigene families [61], [62], [64].

### Ancestry of Nucleotide Sequence Polymorphism in the *HMA4* Genomic Region

The evolutionary events reflected in the profile of nucleotide sequence diversity across the *HMA4* region of *A. halleri* occurred concurrent with or after the divergence from the *A. lyrata* lineage. Whereas nucleotide sequence diversity within *A. halleri* ssp. *halleri* was not positively correlated with the genetic divergence from *A. lyrata* across the *HMA4* region (Figure S7A), we detected shared ancestry of nucleotide sequence diversity profiles in the two subspecies of *A. halleri*, ssp. *halleri* and ssp. *gemmifera*. This is supported by a positive correlation between inter-subspecies sequence divergence and sequence diversity π within ssp. *halleri* (Figure S7B), by the grouping of ssp. *gemmifera* alleles among ssp. *halleri* alleles in genealogies (Figures 2, S1D, S3, S4), and by shared polymorphisms among the two *A. halleri* subspecies in the coding sequences of *HMA4* genes (Figure S6) as well as downstream of *HMA4-2* (S8) (Figure S5D). These findings also indicate that our sampling captured a large proportion of sequence diversity within *A. halleri*, which was further confirmed by a larger genetic diversity of *A. halleri* within collection sites than between collection sites or between regional subgroups of collection sites according to analyses of molecular variance (AMOVA) (Table S5).

Our results support two consecutive duplications of *HMA4* with or after the split of the *lyrata* and *halleri* lineages, which was estimated at between 2 mio. years ago according to sequence divergence [66] and around 0.34 mio. years ago according to approximate Bayesian computation [43]. Previous estimates of the timing of *HMA4* duplication events 0.36 and 0.25 mio. years ago, respectively, are likely to require downward adjustment as they were based on single *A. halleri* sequences for each of the three gene copies and did not take into account EGC [43].

### Conclusion

Enhanced *HMA4* gene product dosage is known to functionally underlie the environmental adaptations of heavy metal hyperaccumulation and hypertolerance in the wild plant *A. halleri* [8]. Here, we detect positive selection in *HMA4* promoter regions of *A. halleri*, incurred by either activating *cis*-regulatory mutations or gene copy number expansion of *HMA4*, and likely by both. Furthermore, we identify ectopic gene conversion to effect the concerted evolution of paralogous *HMA4* coding sequences, a finding that adds unexpected complexity to the profile of sequence polymorphism. We expect that, together, our results coin a class of multi-copy genes associated not only with instances of environmental adaptation in plants [6], [51], [67], but also more generally with eukaryotic adaptation [29], [32], [36], [37], [68]. Thus, this work will stimulate the development of crop breeding strategies based on gene copy number variation [34], [69]. In the future, complex profiles of nucleotide sequence polymorphism, as exemplified by the *HMA4* region of *A. halleri*, will deserve designated attention in advanced targeted studies as well as in large-scale genome scanning approaches [2], [3], [70]. Subsequent to gene duplication events [27], alongside neo- and sub-functionalization, selection for more of the same gene product is of higher evolutionary relevance than previously appreciated [26], [31], [32], [71].

## Materials and Methods

### Plant and Soil Sampling, Processing and Multi-Element Analyses

Leaf tissues and soil samples were collected in the field from 18 randomly selected *A. halleri* ssp. *halleri* individuals at 7 European sites (Table 1). A minimum distance of 2 m was kept between sampled individuals to avoid sampling clones because *A. halleri* is stoloniferous. From a subset of collected genotypes, clones were propagated vegetatively and maintained in a greenhouse. For element analysis by Inductively-Coupled Plasma Atomic Emission Spectrometry (ICP-AES), leaf material was washed with ultrapure water and dried at room temperature (RT) for >1 week, followed by processing of samples and measurements as described [11], [12]. For the determination of extractable soil metal concentrations, soil cores were taken down to 0.05 m depth within 0.1 m distance from each individual. Three g of air-dried, sieved soil (2 mm particle size) were extracted in 25 ml of 0.1 M HCl with rotary shaking at 150 rpm at RT for 0.5 h.

For DNA extraction, leaf tissues were frozen in liquid nitrogen immediately after harvest, kept on dry ice for up to 20 h, and stored at −80°C until further processing. Additionally, previously characterized greenhouse-cultivated, clonally propagated genotypes were included in some experiments: the BC1 parent individual from Auby (individual 8.1) [17], [72], individuals 1.1/Lan 3.1 [8], [12] and 1.4/W504 [13] from Langelsheim, and an individual (9.1) of *A. halleri* ssp. *gemmifera* [38] (Table 1).

### Genomic DNA Preparation and DNA Cloning

Genomic DNA was extracted using the DNeasy Plant Mini Kit (Qiagen, Venlo, The Netherlands) from 100 mg of frozen leaf material of each genotype. The thirteen amplicons designed to analyze sequence diversity (S1 to S13) comprised either non-coding (*i.e.*, promoter, UTR and intron) or both non-coding and coding sequences, and were positioned within all three of the *HMA4* gene copies and at loci of increasing distances upstream and downstream of *HMA4* (Figure 1, Figure S1A, Tables S1 and S2). No additional amplicons could be designed in the repeat- and transposon-rich genomic regions between *HMA4* genes [8]. Primer sequences for amplicons S2 to S12 were designed based on available *A. halleri* BAC sequences (Genbank accession numbers EU382073.1 and EU382072.1) (Table S1) [8]. Primer design for S1 and S13 was based on the *Arabidopsis thaliana* and *Arabidopsis lyrata* ssp. *lyrata* genome sequences [39], [73]. In *A. thaliana* and *A. lyrata*, S1 is located 116 and 198 kb upstream of S2, and S13 is located 113 kb and 2.47 Mbp downstream of S12, respectively. Amplicons comprising the 3′-portions of *AhHMA4-1* (S5), *AhHMA4-2* (S7) and *AhHMA4-3* (S10) were simultaneously amplified in each of three independent PCRs using primer pairs that were not copy-specific (Table S1). In contrast, primers for S8 amplified only the 3′-end of *AhHMA4-2* and additional downstream intergenic sequence, taking advantage of copy-specific sequence polymorphisms in the design of the reverse primer (see Figure S5A).

For PCR amplification, 2 µl of genomic DNA were used with GoTaq DNA polymerase (S1, S2, S11 and S13, Promega, Leiden, The Netherlands), Bio-X-Act Long DNA polymerase (S4, S6, Bioline/Gentaur, Brussels, Belgium) or a mix of both enzymes (S3, S9, S12 and S5/S7/S10), the respective primer pairs (0.5 µM each) (Table S1) and dNTPs (200 µM each) (Fermentas, St. Leon-Rot, Germany) in a final volume of 25 µl, the latter enzyme allowing more efficient amplification. PCR reactions were carried out as follows: 3 min at 95°C, followed by 30–32 cycles of 30 s at 95°C, 30 s at 58°C, 1 min per kb at 70–72°C, and a final extension step of 7 min at 70–72°C. PCR products were gel-purified and cloned into the pGEM-T easy vector (Promega, Leiden, The Netherlands) before transformation of *E. coli* DH5α.

### Sequencing, Sequence Assembly and Assignment of Consensi

In order to ensure with high probability that both alleles were sampled in heterozygous individuals through DNA sequencing, plasmid DNA was isolated from overnight cultures of at least eight independent bacterial colonies per amplicon and genotype, 20 clones for S6 and a total of 56 clones for S5/S7/S10, respectively, before sequencing of inserts by the Sanger method on an ABI 3730xl automated sequencer (Applied Biosystems, Darmstadt, Germany) using vector-specific and additional locus-specific primers when required (Table S1). For two individuals, 48 additional clones from two further independent PCRs were sequenced for S5/S7/S10 to resolve remaining sequence ambiguities.

For the S6 amplicon (corresponding to the promoter region of *HMA4-2*), a set of substantially divergent sequences was initially obtained, and, including these, a total of more than the expected maximum of two types of S6 sequences, corresponding to two alleles expected per individual at this single locus, were found in several *A. halleri* individuals. Using a combination of PCR, BAC end sequencing and DNA gel blot analyses of previously isolated *A. halleri* BACs harboring *HMA4* and related sequences [8], the divergent set of sequences was unequivocally attributed to the promoter of *AhHMA2*, which was found to occur in tandem with *AhHMA3* on a BAC clone, but this BAC did not contain any *AhHMA4* coding sequence. *AhHMA2* and *AhHMA3* are orthologs of *AtHMA2* and *AtHMA3* that are located in tandem on chromosome 4 of *A. thaliana* whereas *AtHMA4* is on chromosome 2. *HMA4*, *HMA2* and *HMA3* genes all encode divalent transition metal cation-transporting P_1B_-type ATPases [18], [74].

Sequence assembly was conducted with DNASTAR (DNASTAR Inc., Madison, USA). First, a consensus sequence was generated for each clone. Then, each consensus was compared to all other consensi from the same amplicon in a given individual and to all consensi of the same amplicon from all other individuals to i) correct *Taq* polymerase errors, ii) identify recombinant chimeras that resulted from template switches during PCR amplification [75] and iii) distinguish heterozygous from homozygous loci.

For the 3′-regions of the three *HMA4* gene copies (S5, S7, S10, S8) more than 800 sequences were obtained in total. Among these, sequences were considered to be authentic when the same sequence was observed at least three times from one PCR reaction or in at least two independent PCRs of the same genotype. After removal of chimeras (which accounted for *ca.* 5% of the sequences), a total of 25 consensus sequences were retained for the 3′-regions of *HMA4* gene copies. These consensi were assigned to the three *HMA4* loci taking advantage of i) the copy-specific sequence information for *AhHMA4-2 via* the overlap between S7 and S8 for each individual (see Figure S5A), ii) position information available from two completely sequenced BACs [8], and iii) step-wise inference using a strictly parsimonious approach, similar to the strategy used to solve a SUDOKU in two times three double-blind independent replicates to ensure reproducibility.

### Statistical and Phylogenetic Analyses

After sequence assembly and alignment, DnaSP v5 [76] was used to calculate sequence diversity (π), Tajima's *D* [45], Fu and Li's D* and F* [44], and to conduct other statistical tests of molecular population genetics. MEGA v5 was used for phylogenetic analyses [77]. The ML trees shown throughout were constructed using a general time-reversible model. Rates among sites were assumed to be gamma-distributed with invariant sites, and 5 discrete categories of gamma were used. All sites were used. To estimate bootstrap support for the nodes, 1000 replicates were calculated. Neighbor joining methods yielded essentially the same results for tree branching orders. Genome sequence information from *A. lyrata* ssp. *lyrata* was used as a reference [39]. Network analyses for *HMA4* genes and for S8 were conducted with TCS v1.21 using a connection limit of 95% [78]. Alignment gaps were re-coded with nucleotides to reflect the exact number of mutational steps between sequences in the respective sequence portion. AMOVA (Analysis of Molecular Variance) was carried out with Arlequin 3.5 [79] to compare the contribution of three hierarchical levels to genetic variance: among the geographic regions of the Thuringian Forest (*A. halleri* ssp. *halleri*), the Harz Mountains (*A. halleri* ssp. *halleri*), and Japan (*A. halleri* ssp. *gemmifera*), among geographic collection sites in each of these three regions, and within single geographic collection sites. A total of 1000 permutations were carried out for each locus, with equal weights of 1 for transitions and transversions, and a deletion weight of 0.

### Determination of *HMA4* Gene Copy Number

Quantitative PCR reactions were performed on 5 ng of genomic DNA in 384-well plates with an ABI Prism 7900HT system (Applied Biosystems, Brussels, Belgium) using MESA GREEN qPCR MasterMix (Eurogentec, Liège, Belgium). Mean reaction efficiencies were determined from all reactions for each amplicon (>270 reactions, Table S6) [80] and used to calculate relative gene copy number by normalization with the qBase software [81] using (i) multiple single-copy reference amplicons and (ii) *A. thaliana* genomic DNA (Col-0) as a calibrator [82]. Three single-copy reference amplicons were selected and designed at the 5′- and 3′-ends of the *AhFRD3* gene [12] and in the S13 amplicon (this study), respectively. Their adequacy to normalize gene copy number in our experimental conditions was validated using the geNorm module in qBase (gene stability measure *M* = 0.309, pairwise variation *CV* = 0.121) [83].

### Quantification of Relative *HMA4* Transcript Levels

Fresh cuttings of greenhouse-grown *A. halleri* and *A. lyrata* genotypes were cultivated hydroponically in 0.1× Hoagland solution for about 2 weeks [13]. After rooting, plants were transferred to pots with soil and further grown in a greenhouse with temperature settings of 22°C (day)/20°C (night) and a photoperiod of 16 h light and 8 h dark. Leaf material was harvested twice independently from the same individuals at an interval of eight weeks, immediately frozen in liquid nitrogen and stored at −80°C. *A. thaliana* and *A. lyrata* plants were grown from seeds as described, with harvest of leaves from 6-week-old plants, alongside harvest of *A. halleri* tissues [12]. Total RNA was extracted with TRIzol Reagent (Invitrogen, Karlsruhe, Germany), cDNA was synthesized from 1 µg of DNaseI-treated (Invitrogen) total RNA using oligo-dT and the SuperScript First-Strand Synthesis System (Invitrogen). Quantitative PCR was conducted in 96-well plates with a MyiQ Single Color Real-Time PCR Detection System (Bio-Rad, Munich, Germany) using SYBR Green qPCR Master Mix (Eurogentec, Cologne, Germany). A total of three technical repeats were run per cDNA and primer pair combination. Data were analyzed using iQ5 Optical System Software version 2.0 (Bio-Rad). Relative transcript levels of *HMA4* were calculated by normalization to *EF1α* as a constitutively expressed reference gene [12]. Primers were as follows: *AhHMA4* primers (5′- GCTGCAGCGATGAAAAACAAAC-3′ and 5′-TCCATACAACATCCCGAGGAAC-3′; amplification efficiency: 1.88); *AlHMA4* primers (5′- TGAAGGTGGTGGTGATTGCA-3′ and 5′-CTCTCCACATTGACCAACTTTG-3′; amplification efficiency: 1.90). *AtHMA4* and *EF1α* primers were described earlier [12].

### Accession Numbers

Sequence data are available through EBI (http://www.ebi.ac.uk), accession nos. HE995813 to HE996227.

## Supporting Information

Figure S1 Organization of *HMA4* genomic regions in different *Arabidopsis* species, and sequence relationships among *HMA4* promoters. (A–C) Genomic organization of the *HMA4* region in *A. halleri* ssp. *halleri* (A) shown again from Figure 1, *A. lyrata* ssp. *lyrata* (B), and *A. thaliana* (C). (D) Sequence relationships among S4, S6 and S9 comprising the promoter regions of *HMA4* genes. Given is pair-wise sequence identity (id) over the length of alignment (cov), both given in percent of the size of the shorter sequence of the respective pair, as calculated based on discontiguous megablast (NCBI MEGABLAST using default settings without filtering for low complexity regions). Syntenic genomic *HMA4* regions are shaded in grey (A, B, C). Genes are represented by rectangles (hatched: *HMA4*; vertically striped: truncated *HMA4* pseudogene in *A. lyrata*; open: absent in *A. halleri*), with genes of unknown position shown in grey. Arabidopsis Genome Identifier (AGI) codes and direction of transcription (triangular arrows) are given for a subset of *A. thaliana* genes and corresponding homologous genes in syntenic positions of *A. halleri* and *A. lyrata*. Positions corresponding to the segments S1 to S13 are indicated by vertical arrows (see Figure 1). Note that segments S6 to S10 are unique to *A. halleri*. Color shading in (D) denotes *HMA4* gene copy (*-1*: green; *-2*: yellow; *-3*: orange). Note that in *A. halleri* ssp. *gemmifera* (individual 9.1), primer pairs designed to obtain S6 and S9 (see Table S1) both yielded the same set of four highly similar sequences. Data shown are from this study (*A. halleri* ssp. *gemmifera*), Genbank EU382073.1 and EU382072.1 [8] for *A. halleri* ssp. *halleri*, and the published genome sequences of *A. lyrata* ssp. *lyrata* [39] and *A. thaliana* (http://www.arabidopsis.org). Chr: chromosome; LG: linkage group; *A*: *Arabidopsis*; *t*: *thaliana*, *l*: *lyrata*; *h*: *halleri*; *g*: *gemmifera*.
(TIF)

Figure S2 Comparison of diversity statistics from this study with data published for *A. halleri*. Shown are Tajima's *D* values as a function of average pairwise nucleotide sequence diversity π re-plotted for the loci analyzed in this study (Table S2, Figure 1) and for other loci with publicly available data. Published dataset A is based on 8 to 14 sequences for each of 8 loci from individuals collected in France and the Czech Republic [41], as well as 12 sequences for each of 24 loci from individuals from Stutenkamm/Thuringian Forest (our collection site no. 7) [42]. Dataset B includes 29 nuclear genes, with one sequence from each of 31 individuals collected in France, Italy, Germany, Slovenia, Poland and the Czech Republic, and with π computed from values listed separately for synonymous and non-synonymous positions [43]. Median and 10/90 percentiles of all shown datapoints are given as a red filled circle and red error bars, respectively. Datapoints S5, S7, S8 and S10 from this study, as well as two datapoints of π = 0 [43], are not shown because Tajima's D values cannot be computed.
(TIF)

Figure S3 Phylogenetic trees for additional segments comprising unique sequences in the genomic *HMA4* region of *A. halleri*. Shown are maximum likelihood trees for amplicons (A) S1, (B) S3, (C) S4, (D) S6, (E) S11, and (F) S13. Alleles are named according to *A. halleri* individuals ([collection site].[individual], see Table 1) and are color-coded based on the population of their origin: blue, Harz Mountains (1 to 5); violet, Thuringian Forest (6 and 7, BAC); brown, Auby (8.1); red, *A. halleri* ssp. *gemmifera* (9.1). Published sequences from *A. halleri* BACs were included (Genbank accession numbers EU382073.1 and EU382072.1) [8], and *A. lyrata* ssp. *lyrata* sequences [39] are shown as outgroup (green), where possible. Percentages of bootstrap support (1000 replicates) of a minimum of 75% are given at their corresponding nodes. Branch lengths are scaled by the number of substitutions per site. The datasets were as follows (number of sequences × number of aligned positions in bp): S1: 31×1978 (A); S3: 42×1312 (B); S4: 42×1927 (C); S6: 43×918 (D); S11: 38×1538 (E); and S13: 39×1739 (F). Asterisks (*) denote the second alleles that were inferred in individuals from which only a single sequence was obtained and which were thus concluded to be homozygous. Note that in *A. halleri* ssp. *gemmifera* (individual 9.1), primer pairs designed to obtain S6 and S9 (see Table S1) both yielded the same set of four highly similar sequences (see Figure S1D). As the *A. halleri* ssp. *halleri* S6 amplicon is shorter than S9, the corresponding sequences (^†^) from *A. halleri ssp. gemmifera* were truncated in the alignment used to infer the S6 tree (E). The phylogenetic trees for segments S2, S9 and S12 are presented in Figure 2.
(TIF)

Figure S4 Phylogenetic tree for segments corresponding to the almost identical 3′-portions of the coding sequences of *HMA4-1*, *-2*, and *-3* of *A. halleri*. Shown is a maximum likelihood tree for segments S5, S7 and S10 (see also Figure 4). The analysis included 25 consensi (h01 to h25) for S5/S7/S10 of *A. halleri* ssp. *halleri*, five distinct *HMA4* sequences obtained from *A. halleri* ssp. *gemmifera* (individual 9.1), and the corresponding segments from two copies of *A. lyrata ssp. lyrata HMA4* and *A. thaliana HMA4*. Sequencing showed that all S5, S7 and S10 were jointly amplified by PCR with each of the primer combinations (Table S1). Post-sequencing assignment (see Materials and Methods) to the three *HMA4* gene copies of *A. halleri* is given in color (green: *HMA4-1*, yellow: *HMA4-2*, and orange: *HMA4-3*). Consensi containing alleles assigned to different *HMA4* gene copies or assigned ambiguously appear in a combination of colors (see Figure 4). Percentages of bootstrap support (1000 replicates) of a minimum of 75% are given at their corresponding nodes. Branch lengths are scaled by the number of substitutions per site. The dataset was 30×1252 (number of sequences×number of aligned positions in bp). ^§^Truncated *HMA4* pseudogene copy in *A. lyrata* ssp. *lyrata* (see Figure S1B).
(TIF)

Figure S5 Ectopic gene conversion between non-coding sequences in the genomic *HMA4* region of *A. halleri*. (A) Organization of the S8 amplicon. The S8 amplicon was uniquely amplified by PCR; portions comprising repeated sequence stretches present in several, almost identical copies in the *HMA4* genomic region are represented in red (see Figure 1), and portions of unique sequence in black. Only the 5′- and 3′-ends of S8 were sequenced: The 5′-end corresponds to *HMA4-2* coding sequence and overlaps with S7. The sequenced 3′-end of the amplicon was designated segment S8 and used in all analyses of sequence data (B, see also Figure 1, Table S2). Numbers indicate length in bp based on BAC contig. (B) Maximum likelihood tree for segment S8. Alleles are named according to *A. halleri* individuals ([collection site].[individual], see Table 1) and are color-coded based on the population of their origin: blue, Harz Mountains (1 to 5); violet, Thuringian Forest (6 and 7, BAC); red, *A. halleri* ssp. *gemmifera* (9.1). A published sequence was included (Genbank accession number EU382072.1) [8]. Percentages of bootstrap support (1000 replicates) did not reach a minimum of 75%. Branch lengths are scaled by the number of substitutions per site. The dataset was 39×501 (number of sequences × number of aligned positions in bp). Asterisks (*) denote alleles that were inferred in individuals from which only a single sequence was obtained and which were concluded to be homozygous. (C) Network analysis of consensus sequences of the repeated 5′-portion of segment S8, and, (D) polymorphic positions in consensus sequences, additionally including those from *A. halleri* ssp. *gemmifera*. Each node represents one mutational step (C). Node size corresponds to the number of alleles per consensus. Alleles constituting the respective consensus sequence (h1 to h6) are specified ([collection site].[individual], see Table 1). SNPs and indels present in single (blue fonts) or multiple (red fonts) consensi are highlighted (D). Note that the consensus h3 is shared between the position downstream of *HMA4-1* (BAC contig) [8] and several alleles of S8 (exclusively downstream of *HMA4-2*) obtained in this study. The analyses (C,D) were conducted based on an alignment of partial S8 sequences downstream of the *HMA4-2* coding sequence comprising 240 positions (yellow, positions 155593-155801 in the *HMA4* BAC contig of EU382072.1 and EU382073.1; 209 bp, shown in red in (A)) that are repeated downstream of *HMA4-1* (green, positions 73657–73865 in the *HMA4* BAC contig, 98.1% nucleotide sequence identity) and downstream of *HMA4-3* (orange, positions 211208–211416 in the *HMA4* BAC contig, 99.0% nucleotide sequence identity), respectively. B in (C): BAC.
(TIF)

Figure S6 Polymorphic positions in consensus sequences of partial *HMA4* coding regions of both *A. halleri* ssp. *halleri* and *A. halleri* ssp. *gemmifera*. Consensus sequences (h01 to h25 in ssp. *halleri* ¸ shown again from Figure 4, and *A. gem*. A to E in ssp. *gemmifera*) were assigned to *HMA4*-*1* (green, S5), -*2* (yellow, S7) or -*3* (orange, S10). Positions given in header row refer to the alignment of consensi. SNPs present in single (blue fonts) or multiple (red fonts) consensi are highlighted, as well as the only SNP that distinguishes all ssp. *gemmifera* from all ssp. *halleri* sequences (position 408, blue fill). For indels, each allele is shown in a different color.
(TIF)

Figure S7 Relationships between inter-(sub)species nucleotide sequence divergence and sequence diversity in *A. halleri* ssp. *halleri*. Shown is nucleotide sequence divergence (K) between (A) *A. lyrata* ssp. *lyrata* or (B) *A. halleri* ssp. *gemmifera* and *A. halleri* ssp. *halleri*, as a function of average pair-wise nucleotide sequence diversity π within *A. halleri* ssp. *halleri*. Shown values are listed in tables on the right. K was estimated using the Jukes and Cantor correction. Note that slight differences of π values between (A) and (B) result from differences in alignments. In the comparison of *A. halleri* ssp. *halleri* with *A. halleri* ssp. *gemmifera* (B), the correlation analysis was conducted including and excluding S6 and S9, respectively, because of an apparent ectopic gene conversion event among these segments in the lineage of *A. halleri* ssp. *gemmifera* individual 9.1 (see Figures 2, S1 and S3). Correlation coefficient (*R* ^2^), degrees of freedom (*df*), *F*-ratios (*F*) and statistical significance (*P*-values, *P*) are given in each panel (A, B).
(TIF)

Table S1 Information on DNA amplicons and on the sequencing of cloned amplicons.
(PDF)

Table S2 Summary of statistics on nucleotide sequence diversity as shown in Figure 1.
(PDF)

Table S3 Identification within amplicons of repeated sequence stretches present in several copies in the *HMA4* genomic region.
(PDF)

Table S4 Robustness of π estimation.
(PDF)

Table S5 Analyses of Molecular variance (AMOVA) in *A. halleri*.
(PDF)

Table S6 Sequences and reaction efficiencies of primer pairs used for quantitative PCR determination of genomic copy number.
(PDF)

## References

[1] Hoekstra HE, Coyne JA (2007) The locus of evolution: evo devo and the genetics of adaptation. Evolution 61: 995–1016. 17492956 10.1111/j.1558-5646.2007.00105.x

[2] Mitchell-Olds T, Willis JH, Goldstein DB (2007) Which evolutionary processes influence natural genetic variation for phenotypic traits? Nat Rev Genet 8: 845–856. 17943192 10.1038/nrg2207

[3] Stinchcombe JR, Hoekstra HE (2008) Combining population genomics and quantitative genetics: finding the genes underlying ecologically important traits. Heredity 100: 158–170. 17314923 10.1038/sj.hdy.6800937

[4] Antonovics J, Bradshaw AD, Turner RG (1971) Heavy metal tolerance in plants. Adv Ecol Res 7: 1–85.

[5] Ernst WHO (1974) Schwermetallvegetationen der Erde. Stuttgart, Germany: Gustav Fischer Verlag.

[6] Krämer U (2010) Metal hyperaccumulation in plants. Annu Rev Plant Biol 61: 517–534. 20192749 10.1146/annurev-arplant-042809-112156

[7] Verbruggen N, Hermans C, Schat H (2009) Molecular mechanisms of metal hyperaccumulation in plants. New Phytol 181: 759–776. 19192189 10.1111/j.1469-8137.2008.02748.x

[8] Hanikenne M, Talke IN, Haydon MJ, Lanz C, Nolte A, et al (2008) Evolution of metal hyperaccumulation required *cis*-regulatory changes and triplication of *HMA4* . Nature 453: 391–395. 18425111 10.1038/nature06877

[9] Boyd R (2010) Elemental defenses of plants by metals. Nature Education Knowledge 1: 6.

[10] Clemens S (2001) Molecular mechanisms of plant metal tolerance and homeostasis. Planta 212: 475–486. 11525504 10.1007/s004250000458

[11] Becher M, Talke IN, Krall L, Krämer U (2004) Cross-species microarray transcript profiling reveals high constitutive expression of metal homeostasis genes in shoots of the zinc hyperaccumulator *Arabidopsis halleri* . Plant J 37: 251–268. 14690509 10.1046/j.1365-313x.2003.01959.x

[12] Talke IN, Hanikenne M, Krämer U (2006) Zinc-dependent global transcriptional control, transcriptional deregulation, and higher gene copy number for genes in metal homeostasis of the hyperaccumulator *Arabidopsis halleri* . Plant Physiol 142: 148–167. 16844841 10.1104/pp.105.076232 PMC1557598

[13] Weber M, Harada E, Vess C, Roepenack-Lahaye EV, Clemens S (2004) Comparative microarray analysis of *Arabidopsis thaliana* and *Arabidopsis halleri* roots identifies nicotianamine synthase, a ZIP transporter and other genes as potential metal hyperaccumulation factors. Plant J 37: 269–281. 14690510 10.1046/j.1365-313x.2003.01960.x

[14] Dräger DB, Desbrosses-Fonrouge AG, Krach C, Chardonnens AN, Meyer RC, et al (2004) Two genes encoding *Arabidopsis halleri* MTP1 metal transport proteins co-segregate with zinc tolerance and account for high *MTP1* transcript levels. Plant J 39: 425–439. 15255871 10.1111/j.1365-313X.2004.02143.x

[15] Deinlein U, Weber M, Schmidt H, Rensch S, Trampczynska A, et al (2012) Elevated nicotianamine levels in *Arabidopsis halleri* roots play a key role in zinc hyperaccumulation. Plant Cell 24: 708–723. 22374395 10.1105/tpc.111.095000 PMC3315242

[16] Lin YF, Liang HM, Yang SY, Boch A, Clemens S, et al (2009) Arabidopsis IRT3 is a zinc-regulated and plasma membrane localized zinc/iron transporter. New Phytol 182: 392–404. 19210716 10.1111/j.1469-8137.2009.02766.x

[17] Courbot M, Willems G, Motte P, Arvidsson S, Roosens N, et al (2007) A major QTL for Cd tolerance in *Arabidopsis halleri* co-localizes with *HMA4*, a gene encoding a heavy metal ATPase. Plant Physiol 144: 1052–1065. 17434989 10.1104/pp.106.095133 PMC1914159

[18] Hussain D, Haydon MJ, Wang Y, Wong E, Sherson SM, et al (2004) P-type ATPase heavy metal transporters with roles in essential zinc homeostasis in Arabidopsis. Plant Cell 16: 1327–1339. 15100400 10.1105/tpc.020487 PMC423219

[19] Willems G, Dräger DB, Courbot M, Gode C, Verbruggen N, et al (2007) The genetic basis of zinc tolerance in the metallophyte *Arabidopsis halleri* ssp. *halleri* (Brassicaceae): An analysis of quantitative trait loci. Genetics 176: 659–674. 17409091 10.1534/genetics.106.064485 PMC1893047

[20] Willems G, Frérot H, Gennen J, Salis P, Saumitou-Laprade P, et al (2010) Quantitative trait loci analysis of mineral element concentrations in an *Arabidopsis halleri*×*Arabidopsis lyrata petraea* F_2_ progeny grown on cadmium-contaminated soil. New Phytol 187: 368–379. 20487315 10.1111/j.1469-8137.2010.03294.x

[21] Frérot H, Faucon MP, Willems G, Gode C, Courseaux A, et al (2010) Genetic architecture of zinc hyperaccumulation in *Arabidopsis halleri*: the essential role of QTL×environment interactions. New Phytol 187: 355–367. 20487314 10.1111/j.1469-8137.2010.03295.x

[22] Papoyan A, Kochian LV (2004) Identification of *Thlaspi caerulescens* genes that may be involved in heavy metal hyperaccumulation and tolerance. Characterization of a novel heavy metal transporting ATPase. Plant Physiol 136: 3814–3823. 15516513 10.1104/pp.104.044503 PMC527178

[23] O'Lochlainn S, Bowen HC, Fray RG, Hammond JP, King GJ, et al (2011) Tandem quadruplication of *HMA4* in the zinc (Zn) and cadmium (Cd) hyperaccumulator *Noccaea caerulescens* . PloS One 6: e17814. 21423774 10.1371/journal.pone.0017814 PMC3053397

[24] Shahzad Z, Gosti F, Frérot H, Lacombe E, Roosens N, et al (2010) The five AhMTP1 zinc transporters undergo different evolutionary fates towards adaptive evolution to zinc tolerance in *Arabidopsis halleri* . PLoS Genet 6: e1000911. 20419142 10.1371/journal.pgen.1000911 PMC2855318

[25] Mirouze M, Sels J, Richard O, Czernic P, Loubet S, et al (2006) A putative novel role for plant defensins: a defensin from the zinc hyper-accumulating plant, *Arabidopsis halleri*, confers zinc tolerance. Plant J 47: 329–342. 16792695 10.1111/j.1365-313X.2006.02788.x

[26] Ohno S (1970) Evolution by gene duplication. New York: Springer.

[27] Lipinski KJ, Farslow JC, Fitzpatrick KA, Lynch M, Katju V, et al (2011) High spontaneous rate of gene duplication in *Caenorhabditis elegans* . Curr Biol 21: 306–310. 21295484 10.1016/j.cub.2011.01.026 PMC3056611

[28] Lynch M, Sung W, Morris K, Coffey N, Landry CR, et al (2008) A genome-wide view of the spectrum of spontaneous mutations in yeast. Proc Natl Acad Sci U S A 105: 9272–9277. 18583475 10.1073/pnas.0803466105 PMC2453693

[29] Chen JM, Cooper DN, Chuzhanova N, Ferec C, Patrinos GP (2007) Gene conversion: mechanisms, evolution and human disease. Nat Rev Genet 8: 762–775. 17846636 10.1038/nrg2193

[30] Michaelson JJ, Shi Y, Gujral M, Zheng H, Malhotra D, et al (2012) Whole-genome sequencing in autism identifies hot spots for *de novo* germline mutation. Cell 151: 1431–1442. 23260136 10.1016/j.cell.2012.11.019 PMC3712641

[31] Lynch M, Conery JS (2000) The evolutionary fate and consequences of duplicate genes. Science 290: 1151–1155. 11073452 10.1126/science.290.5494.1151

[32] Innan H, Kondrashov F (2010) The evolution of gene duplications: classifying and distinguishing between models. Nat Rev Genet 11: 97–108. 20051986 10.1038/nrg2689

[33] Michelmore RW, Meyers BC (1998) Clusters of resistance genes in plants evolve by divergent selection and a birth-and-death process. Genome Res 8: 1113–1130. 9847076 10.1101/gr.8.11.1113

[34] Sutton T, Baumann U, Hayes J, Collins NC, Shi BJ, et al (2007) Boron-toxicity tolerance in barley arising from efflux transporter amplification. Science 318: 1446–1449. 18048688 10.1126/science.1146853

[35] Maron LG, Guimaraes CT, Kirst M, Albert PS, Birchler JA, et al (2013) Aluminum tolerance in maize is associated with higher *MATE1* gene copy number. Proc Natl Acad Sci U S A 110: 5241–5246. 23479633 10.1073/pnas.1220766110 PMC3612656

[36] Perry GH, Dominy NJ, Claw KG, Lee AS, Fiegler H, et al (2007) Diet and the evolution of human amylase gene copy number variation. Nat Genet 39: 1256–1260. 17828263 10.1038/ng2123 PMC2377015

[37] Kondrashov FA (2012) Gene duplication as a mechanism of genomic adaptation to a changing environment. Phil Trans R Soc B 279: 5048–5057. 10.1098/rspb.2012.1108 PMC3497230 22977152

[38] Kubota H, Takenaka C (2003) *Arabis gemmifera* is a hyperaccumulator of Cd and Zn. Int J Phytoremediation 5: 197–201. 14750427 10.1080/713779219

[39] Hu TT, Pattyn P, Bakker EG, Cao J, Cheng JF, et al (2011) The *Arabidopsis lyrata* genome sequence and the basis of rapid genome size change. Nat Genet 43: 476–481. 21478890 10.1038/ng.807 PMC3083492

[40] Barton NH (2000) Genetic hitchhiking. Phil Trans R Soc B 355: 1553–1562. 11127900 10.1098/rstb.2000.0716 PMC1692896

[41] Ramos-Onsins SE, Stranger BE, Mitchell-Olds T, Aguade M (2004) Multilocus analysis of variation and speciation in the closely related species *Arabidopsis halleri* and *A. lyrata* . Genetics 166: 373–388. 15020431 10.1534/genetics.166.1.373 PMC1470697

[42] Heidel AJ, Ramos-Onsins SE, Wang WK, Chiang TY, Mitchell-Olds T (2010) Population history in *Arabidopsis halleri* using multilocus analysis. Mol Ecol 19: 3364–3379. 20670364 10.1111/j.1365-294X.2010.04761.x PMC2921003

[43] Roux C, Castric V, Pauwels M, Wright SI, Saumitou-Laprade P, et al (2011) Does speciation between *Arabidopsis halleri* and *Arabidopsis lyrata* coincide with major changes in a molecular target of adaptation? PloS One 6: e26872. 22069475 10.1371/journal.pone.0026872 PMC3206069

[44] Fu YX, Li WH (1993) Statistical tests of neutrality of mutations. Genetics 133: 693–709. 8454210 10.1093/genetics/133.3.693 PMC1205353

[45] Tajima F (1989) Statistical method for testing the neutral mutation hypothesis by DNA polymorphism. Genetics 123: 585–595. 2513255 10.1093/genetics/123.3.585 PMC1203831

[46] Purugganan MD, Fuller DQ (2009) The nature of selection during plant domestication. Nature 457: 843–848. 19212403 10.1038/nature07895

[47] Benovoy D, Drouin G (2009) Ectopic gene conversions in the human genome. Genomics 93: 27–32. 18848875 10.1016/j.ygeno.2008.09.007

[48] Petes TD, Hill CW (1988) Recombination Between Repeated Genes in Microorganisms. Ann Rev Genet 22: 147–168. 3071247 10.1146/annurev.ge.22.120188.001051

[49] Casola C, Conant GC, Hahn MW (2012) Very low rate of gene conversion in the yeast genome. Mol Biol Evol 89: 3817–3826. 22844073 10.1093/molbev/mss192

[50] Gao LZ, Innan H (2004) Very low gene duplication rate in the yeast genome. Science 306: 1367–1370. 15550669 10.1126/science.1102033

[51] Kroymann J, Donnerhacke S, Schnabelrauch D, Mitchell-Olds T (2003) Evolutionary dynamics of an Arabidopsis insect resistance quantitative trait locus. Proc Natl Acad Sci U S A 100: 14587–14592. 14506289 10.1073/pnas.1734046100 PMC304123

[52] Bosch E, Hurles ME, Navarro A, Jobling MA (2004) Dynamics of a human interparalog gene conversion hotspot. Genome Res 14: 835–844. 15123583 10.1101/gr.2177404 PMC479110

[53] Assis R, Kondrashov AS (2012) A strong deletion bias in nonallelic gene conversion. PLoS Genet 8: e1002508. 22359514 10.1371/journal.pgen.1002508 PMC3280953

[54] Casola C, Zekonyte U, Phillips AD, Cooper DN, Hahn MW (2012) Interlocus gene conversion events introduce deleterious mutations into at least 1% of human genes associated with inherited disease. Genome Res 22: 429–435. 22090377 10.1101/gr.127738.111 PMC3290778

[55] Innan H (2002) A method for estimating the mutation, gene conversion and recombination parameters in small multigene families. Genetics 161: 865–872. 12072480 10.1093/genetics/161.2.865 PMC1462133

[56] Innan H (2003) The coalescent and infinite-site model of a small multigene family. Genetics 163: 803–810. 12618415 10.1093/genetics/163.2.803 PMC1462437

[57] Mano S, Innan H (2008) The evolutionary rate of duplicated genes under concerted evolution. Genetics 180: 493–505. 18757936 10.1534/genetics.108.087676 PMC2535699

[58] Teshima KM, Innan H (2012) The coalescent with selection on copy number variants. Genetics 190: 1077–1086. 22174068 10.1534/genetics.111.135343 PMC3296243

[59] Ohta T (1983) On the evolution of multigene families. Theor Popul Biol 23: 216–240. 6612633 10.1016/0040-5809(83)90015-1

[60] Baekgaard L, Mikkelsen MD, Sorensen DM, Hegelund JN, Persson DP, et al (2010) A combined zinc/cadmium sensor and zinc/cadmium export regulator in a heavy metal pump. J Biol Chem 285: 31243–31252. 20650903 10.1074/jbc.M110.111260 PMC2951198

[61] Parniske M, Hammond-Kosack KE, Golstein C, Thomas CM, Jones DA, et al (1997) Novel disease resistance specificities result from sequence exchange between tandemly repeated genes at the Cf-4/9 locus of tomato. Cell 91: 821–832. 9413991 10.1016/s0092-8674(00)80470-5

[62] Kuang H, Caldwell KS, Meyers BC, Michelmore RW (2008) Frequent sequence exchanges between homologs of *RPP8* in Arabidopsis are not necessarily associated with genomic proximity. Plant J 54: 69–80. 18182023 10.1111/j.1365-313X.2008.03408.x

[63] Kuang H, Woo SS, Meyers BC, Nevo E, Michelmore RW (2004) Multiple genetic processes result in heterogeneous rates of evolution within the major cluster disease resistance genes in lettuce. Plant Cell 16: 2870–2894. 15494555 10.1105/tpc.104.025502 PMC527186

[64] Mondragon-Palomino M, Gaut BS (2005) Gene conversion and the evolution of three leucine-rich repeat gene families in *Arabidopsis thaliana* . Mol Biol Evol 22: 2444–2456. 16120808 10.1093/molbev/msi241

[65] Bergelson J, Kreitman M, Stahl EA, Tian D (2001) Evolutionary dynamics of plant *R*-genes. Science 292: 2281–2285. 11423651 10.1126/science.1061337

[66] Koch MA, Matschinger M (2007) Evolution and genetic differentiation among relatives of *Arabidopsis thaliana* . Proc Natl Acad Sci U S A 104: 6272–6277. 17404224 10.1073/pnas.0701338104 PMC1851049

[67] Dassanayake M, Oh DH, Haas JS, Hernandez A, Hong H, et al (2011) The genome of the extremophile crucifer *Thellungiella parvula* . Nat Genet 43: 913–918. 21822265 10.1038/ng.889 PMC3586812

[68] Nair S, Nash D, Sudimack D, Jaidee A, Barends M, et al (2007) Recurrent gene amplification and soft selective sweeps during evolution of multidrug resistance in malaria parasites. Mol Biol Evol 24: 562–573. 17124182 10.1093/molbev/msl185

[69] Cook DE, Lee TG, Guo X, Melito S, Wang K, et al (2012) Copy number variation of multiple genes at *Rhg1* mediates nematode resistance in soybean. Science 338: 1206–1209. 23065905 10.1126/science.1228746

[70] Turner TL, Bourne EC, Von Wettberg EJ, Hu TT, Nuzhdin SV (2010) Population resequencing reveals local adaptation of *Arabidopsis lyrata* to serpentine soils. Nat Genet 42: 260–263. 20101244 10.1038/ng.515

[71] Sugino RP, Innan H (2006) Selection for more of the same product as a force to enhance concerted evolution of duplicated genes. Trends Genet 22: 642–644. 17045359 10.1016/j.tig.2006.09.014

[72] Bert V, MacNair MR, De Laguérie P, Saumitou-Laprade P, Petit D (2000) Zinc tolerance and accumulation in metallicolous and non metallicolous populations of *Arabidopsis halleri* (Brassicaceae). New Phytol 146: 225–233. 10.1046/j.1469-8137.2000.00634.x 33862970

[73] The Arabidopsis Genome Initiative (2000) Analysis of the genome sequence of the flowering plant *Arabidopsis thaliana* . Nature 408: 796–815. 11130711 10.1038/35048692

[74] Morel M, Crouzet J, Gravot A, Auroy P, Leonhardt N, et al (2009) AtHMA3, a P_1B_-ATPase allowing Cd/Zn/Co/Pb vacuolar storage in Arabidopsis. Plant Physiol 149: 894–904. 19036834 10.1104/pp.108.130294 PMC2633814

[75] Bradley RD, Hillis DM (1997) Recombinant DNA sequences generated by PCR amplification. Mol Biol Evol 14: 592–593. 9159937 10.1093/oxfordjournals.molbev.a025797

[76] Librado P, Rozas J (2009) DnaSP v5: a software for comprehensive analysis of DNA polymorphism data. Bioinformatics 25: 1451–1452. 19346325 10.1093/bioinformatics/btp187

[77] Tamura K, Peterson D, Peterson N, Stecher G, Nei M, et al (2011) MEGA5: molecular evolutionary genetics analysis using maximum likelihood, evolutionary distance, and maximum parsimony methods. Mol Biol Evol 28: 2731–2739. 21546353 10.1093/molbev/msr121 PMC3203626

[78] Clement M, Posada D, Crandall KA (2000) TCS: a computer program to estimate gene genealogies. Mol Ecol 9: 1657–1659. 11050560 10.1046/j.1365-294x.2000.01020.x

[79] Excoffier L, Lischer HE (2010) Arlequin suite ver 3.5: a new series of programs to perform population genetics analyses under Linux and Windows. Mol Ecol Resour 10: 564–567. 21565059 10.1111/j.1755-0998.2010.02847.x

[80] Ramakers C, Ruijter JM, Deprez RH, Moorman AF (2003) Assumption-free analysis of quantitative real-time polymerase chain reaction (PCR) data. Neurosci Lett 339: 62–66. 12618301 10.1016/s0304-3940(02)01423-4

[81] Hellemans J, Mortier G, De Paepe A, Speleman F, Vandesompele J (2007) qBase relative quantification framework and software for management and automated analysis of real-time quantitative PCR data. Genome Biol 8: R19. 17291332 10.1186/gb-2007-8-2-r19 PMC1852402

[82] D'Haene B, Vandesompele J, Hellemans J (2010) Accurate and objective copy number profiling using real-time quantitative PCR. Methods 50: 262–270. 20060046 10.1016/j.ymeth.2009.12.007

[83] Vandesompele J, De Preter K, Pattyn F, Poppe B, Van Roy N, et al (2002) Accurate normalization of real-time quantitative RT-PCR data by geometric averaging of multiple internal control genes. Genome Biol 3: RESEARCH0034. 12184808 10.1186/gb-2002-3-7-research0034 PMC126239

