# Supplementary figures and images for: Hard Selective Sweep and Ectopic Gene Conversion in a Gene Cluster Affording Environmental Adaptation
Source: PLoS Genet. 2013 Aug 22;9(8):e1003707. doi: 10.1371/journal.pgen.1003707 (PMC3749932; doi:10.1371/journal.pgen.1003707)

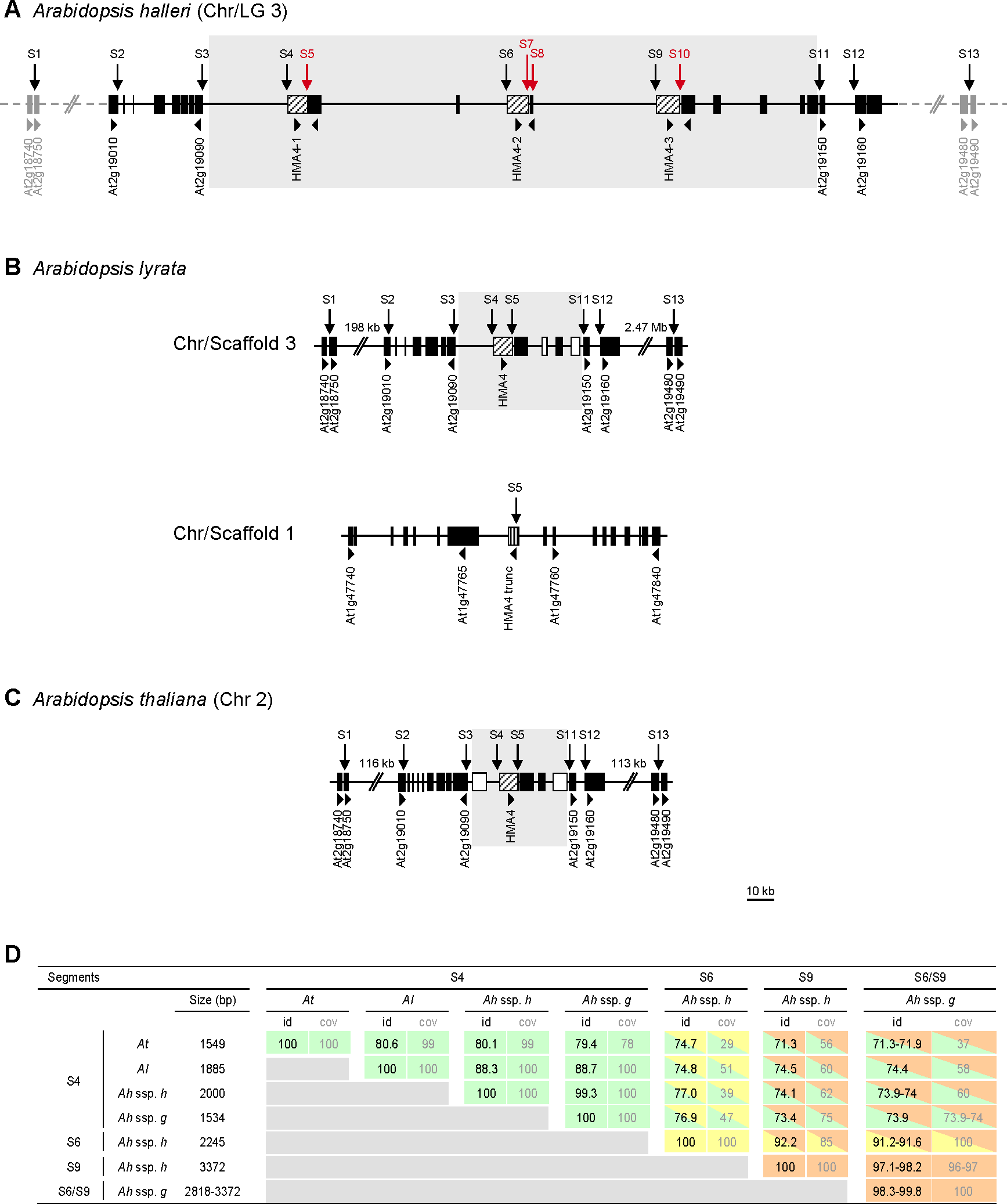

Supplement: Figure S1 — Organization of HMA4 genomic regions in different Arabidopsis species, and sequence relationships among HMA4 promoters. (A–C) Genomic organization of the HMA4 region in A. halleri ssp. halleri (A) shown again from Figure 1, A. lyrata ssp. lyrata (B), and A. thaliana (C). (D) Sequence relationships among S4, S6 and S9 comprising the promoter regions of HMA4 genes. Given is pair-wise sequence identity (id) over the length of alignment (cov), both given in percent of the size of the shorter sequence of the respective pair, as calculated based on discontiguous megablast (NCBI MEGABLAST using default settings without filtering for low complexity regions). Syntenic genomic HMA4 regions are shaded in grey (A, B, C). Genes are represented by rectangles (hatched: HMA4; vertically striped: truncated HMA4 pseudogene in A. lyrata; open: absent in A. halleri), with genes of unknown position shown in grey. Arabidopsis Genome Identifier (AGI) codes and direction of transcription (triangular arrows) are given for a subset of A. thaliana genes and corresponding homologous genes in syntenic positions of A. halleri and A. lyrata. Positions corresponding to the segments S1 to S13 are indicated by vertical arrows (see Figure 1). Note that segments S6 to S10 are unique to A. halleri. Color shading in (D) denotes HMA4 gene copy (-1: green; -2: yellow; -3: orange). Note that in A. halleri ssp. gemmifera (individual 9.1), primer pairs designed to obtain S6 and S9 (see Table S1) both yielded the same set of four highly similar sequences. Data shown are from this study (A. halleri ssp. gemmifera), Genbank EU382073.1 and EU382072.1 [8] for A. halleri ssp. halleri, and the published genome sequences of A. lyrata ssp. lyrata [39] and A. thaliana (http://www.arabidopsis.org). Chr: chromosome; LG: linkage group; A: Arabidopsis; t: thaliana, l: lyrata; h: halleri; g: gemmifera. (TIF) [file pgen.1003707.s001.tif]

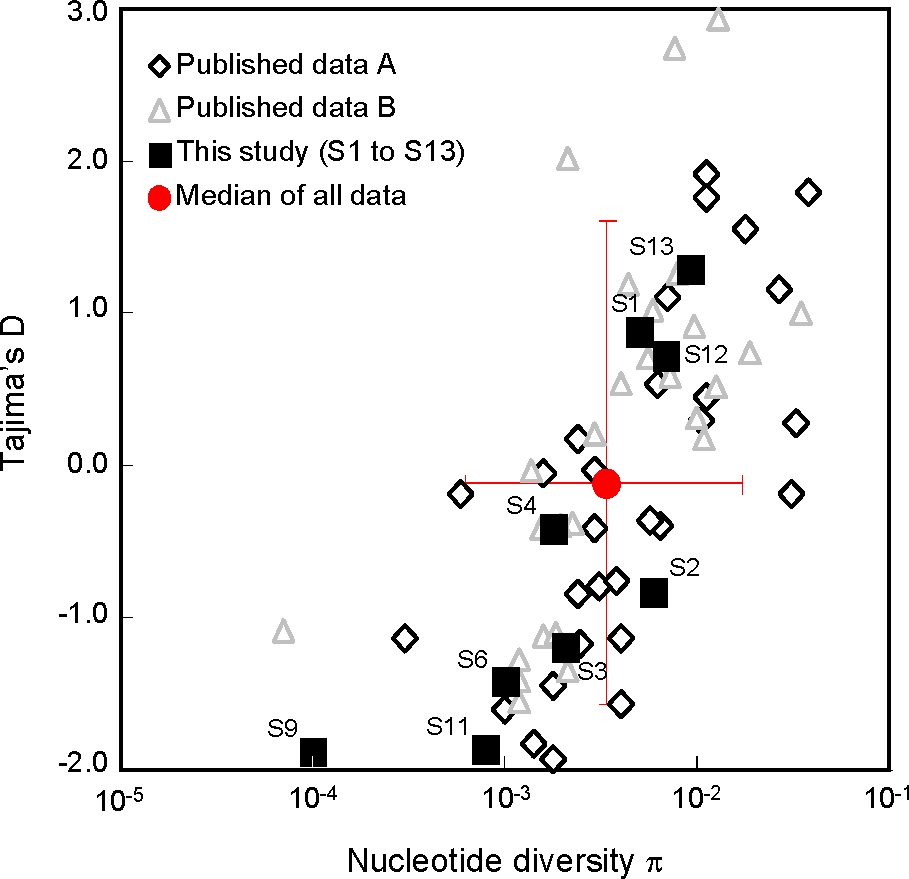

Supplement: Figure S2 — Comparison of diversity statistics from this study with data published for A. halleri. Shown are Tajima's D values as a function of average pairwise nucleotide sequence diversity π re-plotted for the loci analyzed in this study (Table S2, Figure 1) and for other loci with publicly available data. Published dataset A is based on 8 to 14 sequences for each of 8 loci from individuals collected in France and the Czech Republic [41], as well as 12 sequences for each of 24 loci from individuals from Stutenkamm/Thuringian Forest (our collection site no. 7) [42]. Dataset B includes 29 nuclear genes, with one sequence from each of 31 individuals collected in France, Italy, Germany, Slovenia, Poland and the Czech Republic, and with π computed from values listed separately for synonymous and non-synonymous positions [43]. Median and 10/90 percentiles of all shown datapoints are given as a red filled circle and red error bars, respectively. Datapoints S5, S7, S8 and S10 from this study, as well as two datapoints of π = 0 [43], are not shown because Tajima's D values cannot be computed. (TIF) [file pgen.1003707.s002.tif]

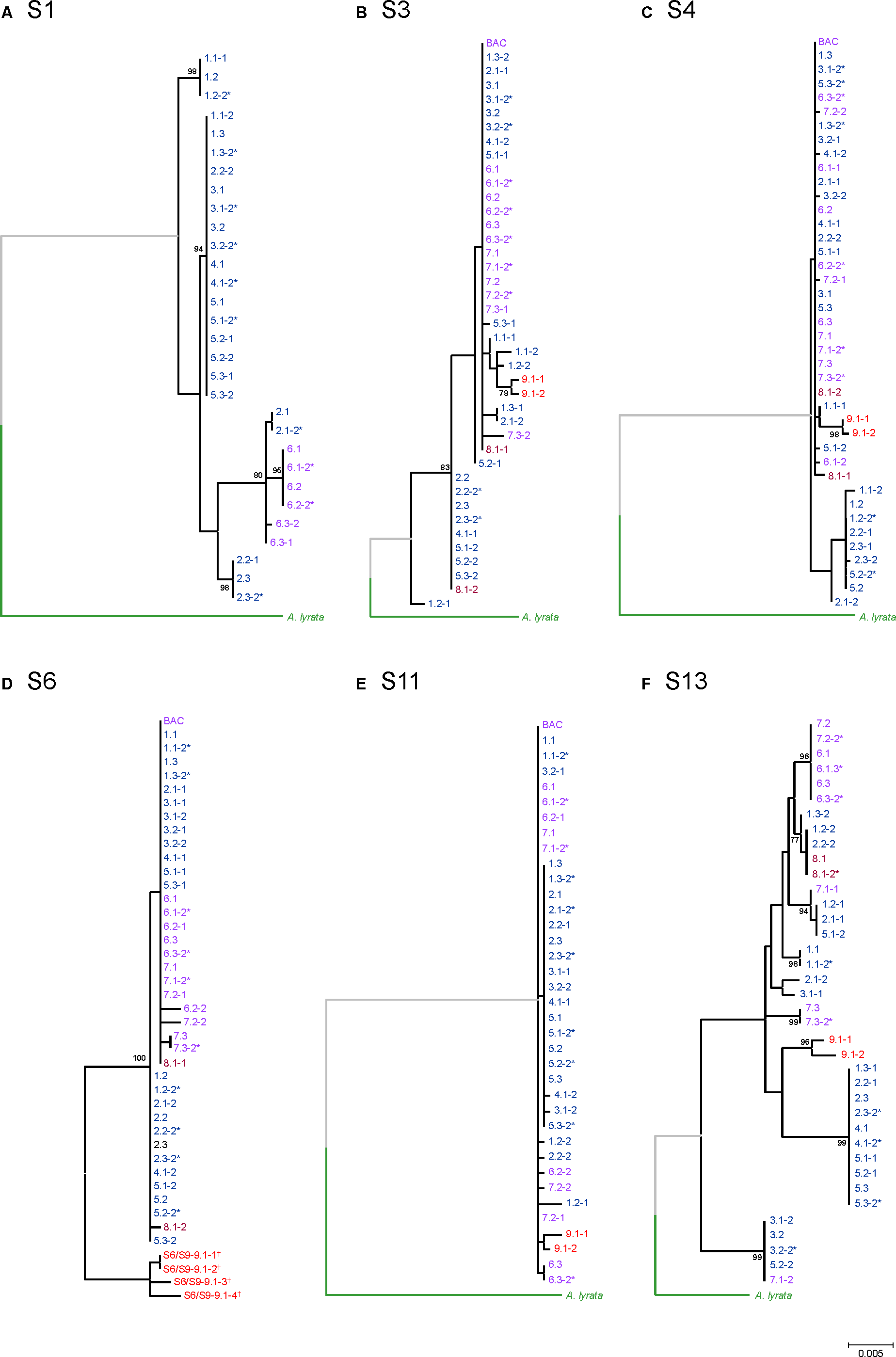

Supplement: Figure S3 — Phylogenetic trees for additional segments comprising unique sequences in the genomic HMA4 region of A. halleri. Shown are maximum likelihood trees for amplicons (A) S1, (B) S3, (C) S4, (D) S6, (E) S11, and (F) S13. Alleles are named according to A. halleri individuals ([collection site].[individual], see Table 1) and are color-coded based on the population of their origin: blue, Harz Mountains (1 to 5); violet, Thuringian Forest (6 and 7, BAC); brown, Auby (8.1); red, A. halleri ssp. gemmifera (9.1). Published sequences from A. halleri BACs were included (Genbank accession numbers EU382073.1 and EU382072.1) [8], and A. lyrata ssp. lyrata sequences [39] are shown as outgroup (green), where possible. Percentages of bootstrap support (1000 replicates) of a minimum of 75% are given at their corresponding nodes. Branch lengths are scaled by the number of substitutions per site. The datasets were as follows (number of sequences × number of aligned positions in bp): S1: 31×1978 (A); S3: 42×1312 (B); S4: 42×1927 (C); S6: 43×918 (D); S11: 38×1538 (E); and S13: 39×1739 (F). Asterisks (*) denote the second alleles that were inferred in individuals from which only a single sequence was obtained and which were thus concluded to be homozygous. Note that in A. halleri ssp. gemmifera (individual 9.1), primer pairs designed to obtain S6 and S9 (see Table S1) both yielded the same set of four highly similar sequences (see Figure S1D). As the A. halleri ssp. halleri S6 amplicon is shorter than S9, the corresponding sequences (†) from A. halleri ssp. gemmifera were truncated in the alignment used to infer the S6 tree (E). The phylogenetic trees for segments S2, S9 and S12 are presented in Figure 2. (TIF) [file pgen.1003707.s003.tif]

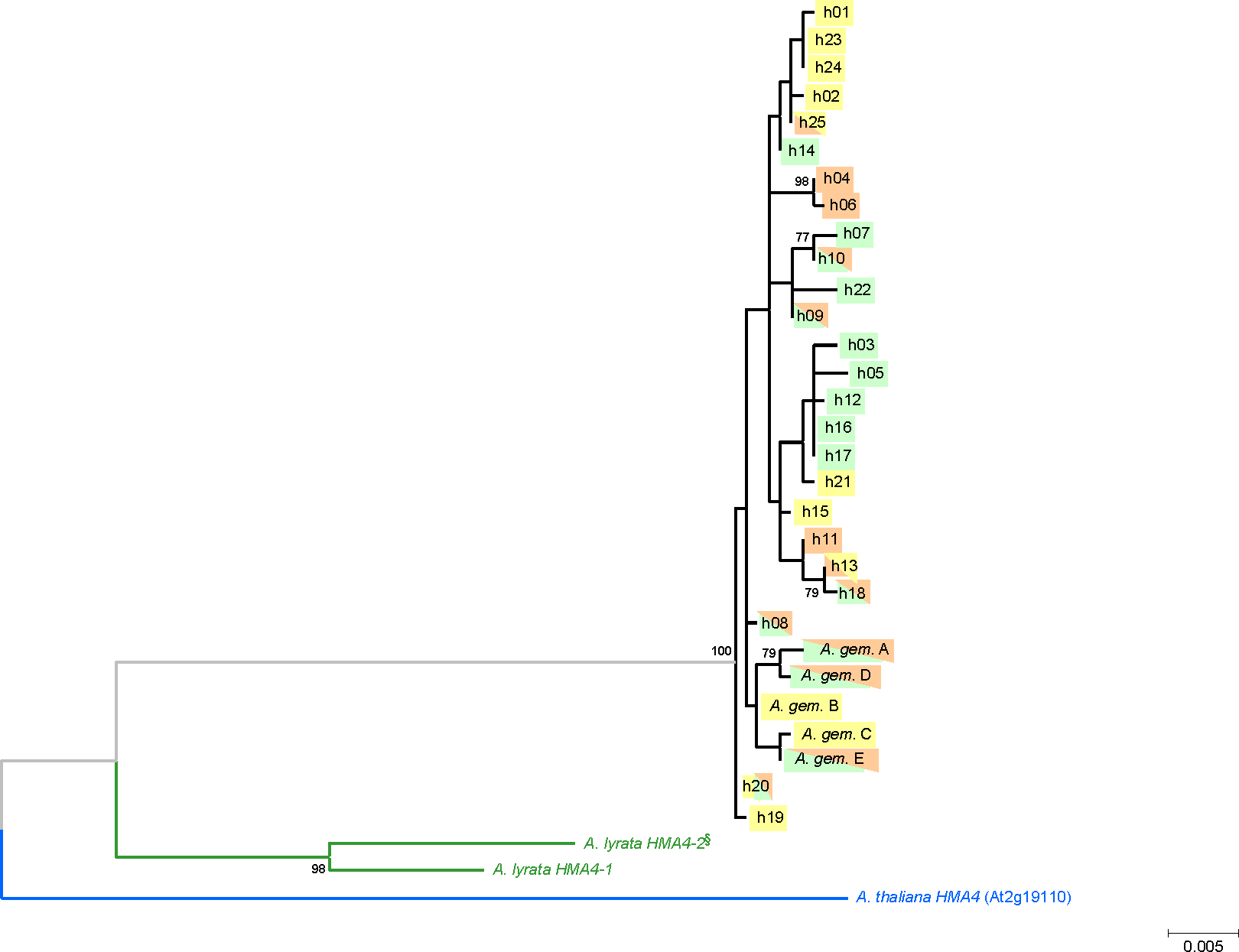

Supplement: Figure S4 — Phylogenetic tree for segments corresponding to the almost identical 3′-portions of the coding sequences of HMA4-1, -2, and -3 of A. halleri. Shown is a maximum likelihood tree for segments S5, S7 and S10 (see also Figure 4). The analysis included 25 consensi (h01 to h25) for S5/S7/S10 of A. halleri ssp. halleri, five distinct HMA4 sequences obtained from A. halleri ssp. gemmifera (individual 9.1), and the corresponding segments from two copies of A. lyrata ssp. lyrata HMA4 and A. thaliana HMA4. Sequencing showed that all S5, S7 and S10 were jointly amplified by PCR with each of the primer combinations (Table S1). Post-sequencing assignment (see Materials and Methods) to the three HMA4 gene copies of A. halleri is given in color (green: HMA4-1, yellow: HMA4-2, and orange: HMA4-3). Consensi containing alleles assigned to different HMA4 gene copies or assigned ambiguously appear in a combination of colors (see Figure 4). Percentages of bootstrap support (1000 replicates) of a minimum of 75% are given at their corresponding nodes. Branch lengths are scaled by the number of substitutions per site. The dataset was 30×1252 (number of sequences×number of aligned positions in bp). §Truncated HMA4 pseudogene copy in A. lyrata ssp. lyrata (see Figure S1B). (TIF) [file pgen.1003707.s004.tif]

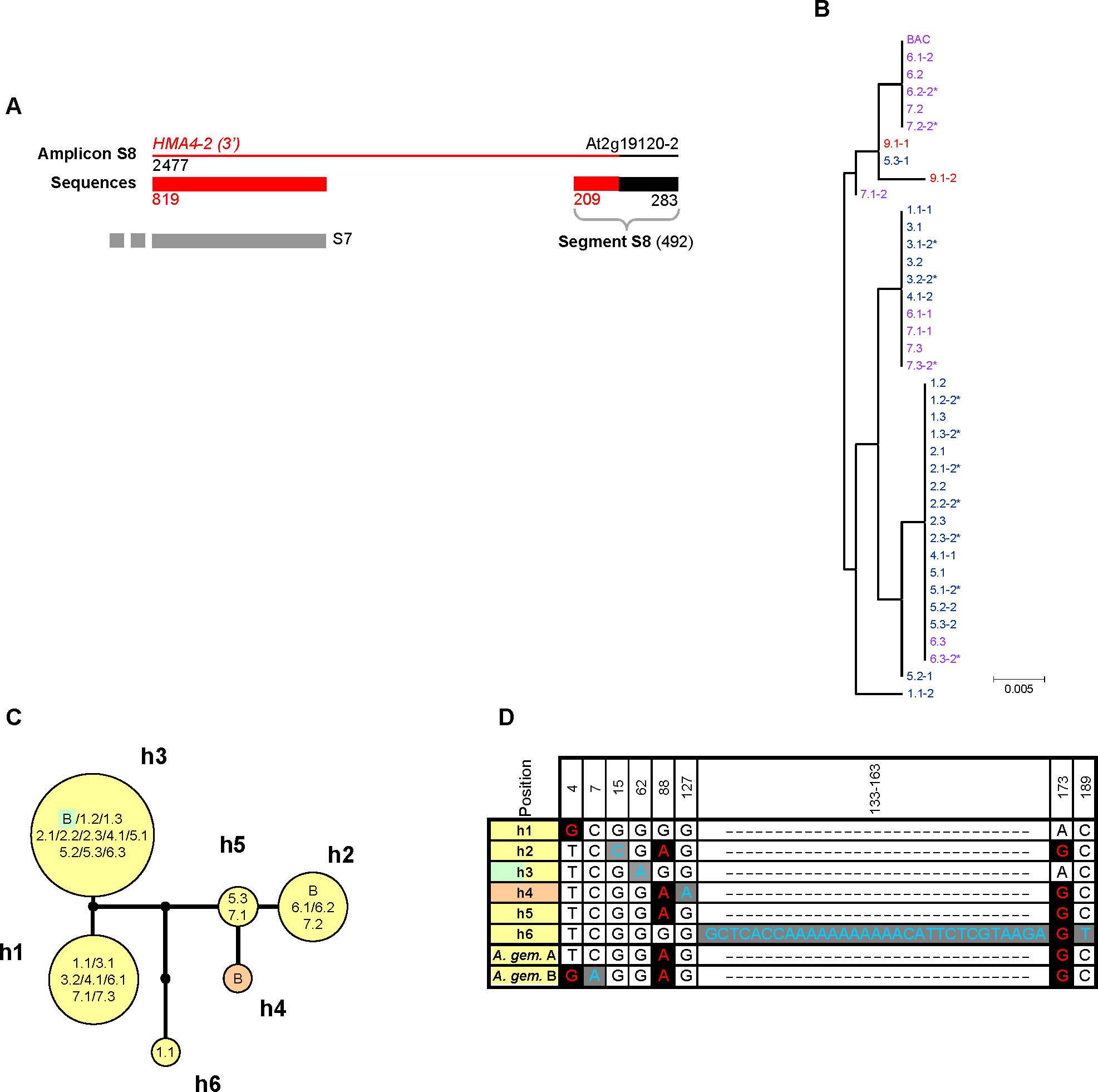

Supplement: Figure S5 — Ectopic gene conversion between non-coding sequences in the genomic HMA4 region of A. halleri. (A) Organization of the S8 amplicon. The S8 amplicon was uniquely amplified by PCR; portions comprising repeated sequence stretches present in several, almost identical copies in the HMA4 genomic region are represented in red (see Figure 1), and portions of unique sequence in black. Only the 5′- and 3′-ends of S8 were sequenced: The 5′-end corresponds to HMA4-2 coding sequence and overlaps with S7. The sequenced 3′-end of the amplicon was designated segment S8 and used in all analyses of sequence data (B, see also Figure 1, Table S2). Numbers indicate length in bp based on BAC contig. (B) Maximum likelihood tree for segment S8. Alleles are named according to A. halleri individuals ([collection site].[individual], see Table 1) and are color-coded based on the population of their origin: blue, Harz Mountains (1 to 5); violet, Thuringian Forest (6 and 7, BAC); red, A. halleri ssp. gemmifera (9.1). A published sequence was included (Genbank accession number EU382072.1) [8]. Percentages of bootstrap support (1000 replicates) did not reach a minimum of 75%. Branch lengths are scaled by the number of substitutions per site. The dataset was 39×501 (number of sequences × number of aligned positions in bp). Asterisks (*) denote alleles that were inferred in individuals from which only a single sequence was obtained and which were concluded to be homozygous. (C) Network analysis of consensus sequences of the repeated 5′-portion of segment S8, and, (D) polymorphic positions in consensus sequences, additionally including those from A. halleri ssp. gemmifera. Each node represents one mutational step (C). Node size corresponds to the number of alleles per consensus. Alleles constituting the respective consensus sequence (h1 to h6) are specified ([collection site].[individual], see Table 1). SNPs and indels present in single (blue fonts) or multiple (red fonts) consensi are highlighted (D [file pgen.1003707.s005.tif]

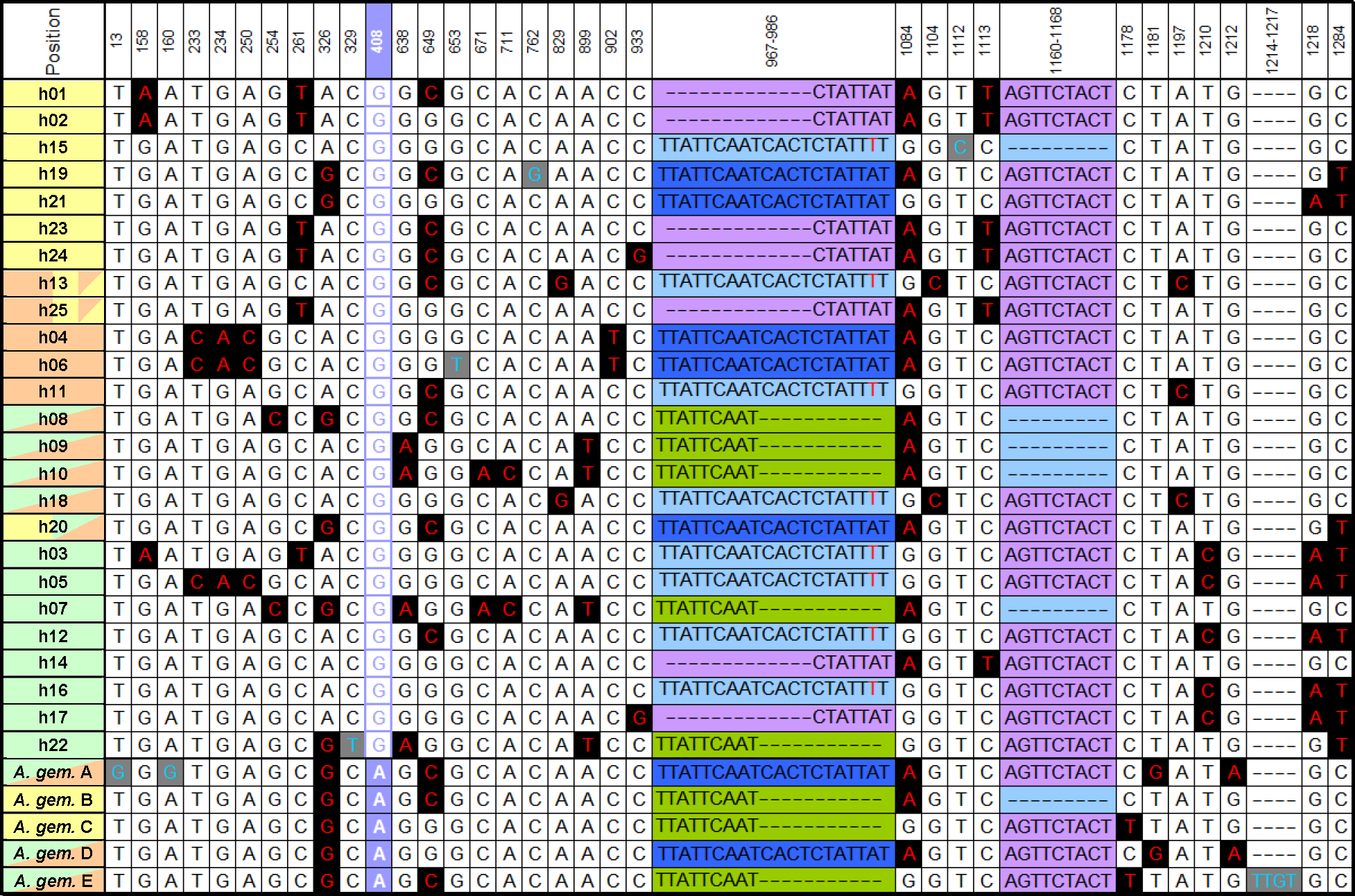

Supplement: Figure S6 — Polymorphic positions in consensus sequences of partial HMA4 coding regions of both A. halleri ssp. halleri and A. halleri ssp. gemmifera. Consensus sequences (h01 to h25 in ssp. halleri ¸ shown again from Figure 4, and A. gem. A to E in ssp. gemmifera) were assigned to HMA4-1 (green, S5), -2 (yellow, S7) or -3 (orange, S10). Positions given in header row refer to the alignment of consensi. SNPs present in single (blue fonts) or multiple (red fonts) consensi are highlighted, as well as the only SNP that distinguishes all ssp. gemmifera from all ssp. halleri sequences (position 408, blue fill). For indels, each allele is shown in a different color. (TIF) [file pgen.1003707.s006.tif]
