# Supplementary material for: Hard Selective Sweep and Ectopic Gene Conversion in a Gene Cluster Affording Environmental Adaptation
Source: PLoS Genet. 2013 Aug 22;9(8):e1003707. doi: 10.1371/journal.pgen.1003707 (PMC3749932; doi:10.1371/journal.pgen.1003707)
Supplement: Table S1 — Information on DNA amplicons and on the sequencing of cloned amplicons. (PDF) [file pgen.1003707.s008.pdf]

Table S1. Information on DNA amplicons and on the sequencing of cloned amplicons.

Amplicon		Composition		Target gene (position)	Annotation[2]	Missing individuals[3]	Sequences of PCR primers used for amplification[4]	
Name	Size (bp)[1]	Coding (bp)[1]	Non-coding (bp)[1]				Forward	Reverse
S1	1593	224	1369	At2g18750 (5')	Calmodulin-binding protein	7.1-7.3, 8.1, 9.1	CAACATAACTCTGATGATGAACACG	CTCGGCGAAGAATCGGTTCC
S2	1451	398	1053	At2g19010 (3')	GDSL-like Lipase/Acylhydrolase	-	GCACACCAAAGATAATGAAGTCACAC	GACTTGCTTAGCGGCTTCCTCG
S3	1305	540	765	At2g19090 (5')	Protein of unknown function	-	CGAAAATAAACATGAAAAGCGAGG	CCAAAACGAAGATGAAGTAGACGC
S4	1885	30	1855	*HMA4-1*$_P$[5] (5')	Heavy Metal ATPase 4	-	GACATTCCACTTTTGGGGGTTTCC	TTTCTCTTCTTCTTTGTTTTGTGACGCC
S5	1419	1301	118	*HMA4-1*[5] (3')	Heavy Metal ATPase 4	-	GCGATGATGATGATGCTGTGGAC	TCAAGCACTCACATGGTGATGGTG
S6	917	30	887	*HMA4-2*$_P$[5] (5')	Heavy Metal ATPase 4	[6]	GCTAAAAACACGCCGATTAAGAAG	TTTCTCTTCTTCTTTGTTTTGTGACGCC
S7	1406	1301	105	*HMA4-2*[5] (3')	Heavy Metal ATPase 4	[6]	GCGATGATGATGATGCTGTGGAC	TCAAGCACTCACATGGTGATGGTG
S8	2477[7]	1346	1131	*HMA4-2*[5] (3')/ At2g19120-2	Heavy Metal ATPase 4	8.1[6]	GACACATCTTACCTGGAGAAG	GCCAGAGTTATATGGTGATATG
S9	2245	30	2215	*HMA4-3*$_P$[5] (5')	Heavy Metal ATPase 4	3.2, 7.3[6]	TTAAAAGGGGTATTGAAAAAGGAGC	TTTCTCTTCTTCTTTGTTTTGTGACGCC
S10	1425	1307	118	*HMA4-3*[5] (3')	Heavy Metal ATPase 4	[6]	GCGATGATGATGATGCTGTGGAC	TCAAGCACTCACATGGTGATGGTG
S11	1326	458	868	At2g19150 (5')	Pectin lyase-like protein	7.3, 8.1	TGAAAATTTCAACAAATAGTGTACCG	GCAGCCACCGCTGGTTTGG
S12	2132	311	1821	At2g19160 (5')	Core-2/I-branching beta-1,6-N-acetylglucosaminyltransferase	-	TAACATGCTTTCCTTTGACGGG	GAACTCTTTTTTATAACACGAGGGG
S13	1694	302	1392	At2g19490 (5')	recA DNA recombination protein	6.2	GGTCAAGCAGGTGAGAGGCCA	CCTCCTTGTTTTTGTGCTTCTGC

[1]Sizes based on *A. halleri* BAC sequences (Genbank accession numbers: EU382073.1, EU382072.1) [8], and on *A. thaliana* genome sequence (Genbank accession numbers: AC005724 and AC005917) for amplicons S1 and S13. Note that primers designed for amplicons S5, S7 and S10 non-specifically cross-amplified sequences from all three of these target gene copies in *A. halleri*; assignment to copies was then done based on S8 and according to Materials and Methods.

[2]TAIR 10 (http://www.arabidopsis.org).

[3]No PCR products were obtained for the given individuals despite attempts using a range of alternative primer pairs and PCR conditions.

[4]Amplicons were sequenced after cloning, using vector-specific primers M13f (CGCCAGGGTTTTCCCAGTCACGAC) and M13r (TCACACAGGAAACAGCTATGAC). When necessary, sequencing was completed using additional gene-specific primers.

[5]All *HMA4* gene copies correspond to At2g19110. $_P$: promoter region.

[6]There is only a single complete *HMA4* gene in the *A. lyrata* genome, which corresponds to *AhHMA4-1* (S4, S5). See also Figure S1.

[7]For S8, only the 3'-end of the amplicon encompassing 492 bp (209 bp coding and 283 bp non-coding[1]) was used as segment S8 in all analyses (see Figure S5A). The 5'-end of amplicon S8 (819 bp[1]) was designed to overlap with S7 and used to identify S7 sequences (see Materials and Methods; Figure S5A).

Red fonts: segments comprising repeated sequence stretches present in several, almost identical copies in the *HMA4* genomic region.
