# Supplementary material for: Hard Selective Sweep and Ectopic Gene Conversion in a Gene Cluster Affording Environmental Adaptation
Source: PLoS Genet. 2013 Aug 22;9(8):e1003707. doi: 10.1371/journal.pgen.1003707 (PMC3749932; doi:10.1371/journal.pgen.1003707)
Supplement: Table S2 — Summary of statistics on nucleotide sequence diversity as shown in Figure 1. (PDF) [file pgen.1003707.s009.pdf]

Table S2. Summary of statistics of nucleotide sequence diversity as shown in Figure 1.

		Segment												
Alleles	Statistics	S1	S2	S3	S4	S5	S6	S7	S8	S9	S10	S11	S12	S13
Sequenced and inferred	π	0.0049	0.0059	0.0021	0.0018	0.0042	0.0010	0.0032	0.0052	0.0001	0.0035	0.0008	0.0069	0.0091
(as shown in Fig. 1)	Tajima's *D*	0.877	-0.835	-1.182	-0.417	n.a.	-1.418	n.a.	n.a.	**-1.886**[§]	n.a.	**-1.863**[§]	0.720	1.294
	Fu and Li's *D**	**1.410**[§]	0.875	-2.283	-1.677	n.a.	-2.442	n.a.	n.a.	**-3.124**[§]	n.a.	**-3.437**[§]	1.082	**1.693**[§]
	Fu and Li's *F**	1.458	0.353	-2.266	-1.490	n.a.	-2.486	n.a.	n.a.	**-3.206**[§]	n.a.	**-3.450**[§]	1.136	**1.845**[§]
	# sites (excluding gaps)	1554	760	1234	1827	1320	841	1327	470	2189	1336	1480	1985	1622
	# haplotypes	7	6	10	13	n.a.	6	n.a.	8	5	n.a.	10	12	11
	N	30	39	39	39	35	39	34	37	35	34	35	39	36
Sequenced only	π	0.0048	0.0077	0.0024	0.0019	/	0.0010	/	0.0055	0.0002	/	0.0010	0.0071	0.0093
	Tajima's *D*	0.392	-0.466	-1.110	-0.491	/	-1.546	/	n.a.	**-1.978**[§]	/	**-1.901**[§]	0.676	1.076
	Fu and Li's *D**	1.164	0.943	-1.981	-1.430	/	**-2.870**[§]	/	n.a.	**-2.716**[§]	/	**-3.404**[§]	1.090	0.905
	Fu and Li's *F**	1.088	0.596	-2.003	-1.331	/	**-2.882**[§]	/	n.a.	**-2.864**[§]	/	**-3.443**[§]	1.126	1.128
	# sites (excluding gaps)	1554	760	1234	1827	/	841	/	470	2189	/	1480	1985	1622
	# haplotypes	7	6	10	13	/	6	/	8	5	/	10	12	11
	N	20	24	30	30	/	29	/	25	22	/	25	33	26
No. of alleles	BAC	-	1	1	1	1	1	1	1	1	1	1	1	-
observed per genotype	1.1	2	2	2	2	1	1	2	2	2	1	1	2	1
	1.2	1	2	2	1	1	1	2	1	1	1	2	2	2
	1.3	1	1	2	1	1	1	2	1	2	2	1	2	2
	2.1	1	1	2	2	1[+]	2	2	1	1	1[+]	1	2	2
	2.2	2	2	1	2	1	1	1	1	1	1	2	2	2
	2.3	1	1	1	2	1	1	1	1	1	1	1	1	1
	3.1	1	1	1	1	2	2	2	1	1	2	2	2	2
	3.2	1	1	1	2	2	2	2	1	-	2	2	2	1
	4.1	1	1	2	2	2	2	2	2	1	2	2	1	1
	5.1	1	1	2	2	2	2	2	1	2	2	1	2	2
	5.2	2	1	2	1	2	1	2	2	1	2	1	1	2
	5.3	2	1	2	1	2	2	2	2	1	2	1	1	1
	6.1	1	1	1	2	2	1	2	2	1	1	1	2	1
	6.2	1	1	1	1	1	2	1	1	1	1	2	1	-
	6.3	2	1	1	1	2	1	2	1	1	2	1	2	1
	7.1	-	1	1	1	2	1	2	2	1	1	1	2	2
	7.2	-	1	1	2	2	2	2	1	2	2	2	1	1
	7.3	-	2	2	1	2	1	2	1	-	2	-	2	1
	8.1	-	1	2	2	2	2	1	-	1	1	-	2	1
	A. halleri ssp. *gemmifera* (9.1)[1]	-	1	2	2	1[+]	4*	2	2	4*	1[+]	2	2	2
	A. lyrata ssp. *lyrata*[1]	1	1	1	1	1	x	x	x	x	x	1	1	1

Values for BAC and *A. lyrata* are based on public data [8,39]. Genotypes for which only a single consensus sequence was obtained were concluded to be homozygous, and the second allele was inferred. Assignment to *HMA4* gene copy of S5, S7 and S10 was done by inference upon the assignment to S7 through anchoring *via* overlapping S8 sequences according to Materials and Methods (marked "/"; [+]: ambiguous assignment to S5 or S10). Grey shade: amplicons in triplicated region (*HMA4* and two downstream genes, see Figure 1). Red fonts: segments comprising repeated sequence stretches present in several, almost identical copies in the *HMA4* genomic region. n.a.: not applicable; sequences did not evolve independently because of ectopic gene conversion, thus precluding tests of molecular population genetics. [§]: $P < 0.05$. -: no PCR product obtained. x: not present in the genome. *: Primer pairs designed to obtain S6 and S9 (see Table S1) both yielded the same set of four highly similar sequences (see Figure S1). [1]Parameters for the *A. halleri* ssp. *gemmifera* and *A. lyrata* ssp. *lyrata* individuals are only shown for comparison; their sequences were not included in the analyses presented above and in Figure 1.
