# Supplementary material for: Hard Selective Sweep and Ectopic Gene Conversion in a Gene Cluster Affording Environmental Adaptation
Source: PLoS Genet. 2013 Aug 22;9(8):e1003707. doi: 10.1371/journal.pgen.1003707 (PMC3749932; doi:10.1371/journal.pgen.1003707)
Supplement: Table S3 — Identification within amplicons of repeated sequence stretches present in several copies in the HMA4 genomic region. (PDF) [file pgen.1003707.s010.pdf]

**Table S3**. Identification within amplicons of repeated sequence stretches present in several copies in the *HMA4* genomic region.

**A**. Sequence similarities between amplified segments and sequences within BAC contig of the *A. halleri* genomic *HMA4* region.

| Amplicon | Sequence identity (%)[1] | Hit length (bp)[1] | Coordinates in amplicon | Coordinates in *HMA4* BAC contig | Annotation |
|---|---|---|---|---|---|
| S2 | 67 | 336 | 1094-1413 r.c. | 22145-22456 | intergenic region At2g19050/At2g19060[2] |
| S3 | - | - | - | - | - |
| S4 | 84 | 310 | 4-298 | 197971-198272 | *AhHMA4-3*$_P$ |
| | 77 | 364 | 1111-1471 | 146010-146336 | *AhHMA4-2*$_P$ |
| S5 | 99 | 1425 | 1-1419 | 208610-210034 | *AhHMA4-3* 3'-end |
| | 99 | 1419 | 1-1419 | 153021-154436 | *AhHMA4-2* 3'-end |
| S6 | 88 | 753 | 224-917 | 200743-201470 | *AhHMA4-3*$_P$ |
| | 77 | 364 | 317-643 | 64760-65120 | *AhHMA4-1*$_P$ |
| S7 | 99 | 1419 | 1-1406 | 71255-72673 | *AhHMA4-1* 3'-end |
| | 99 | 1407 | 1-1394 | 208610-210016 | *AhHMA4-3* 3'-end |
| S8 | 95 | 2263 | 1-2194 | 209197-211416 | intergenic region *AhHMA4-3*/At2g19120-3 |
| | 90 | 2208 | 1-2194 | 71842-73865 | intergenic region *AhHMA4-1*/At2g19120-1 |
| S9 | 80 | 2589 | 43-2245 | 144154-146630 | *AhHMA4-2*$_P$ |
| | 77 | 313 | 1714-1989 | 64809-65120 | *AhHMA4-1*$_P$ |
| S10 | 99 | 1407 | 1-1407 | 71255-72661 | *AhHMA4-1* 3'-end |
| | 99 | 1407 | 1-1407 | 153021-154414 | *AhHMA4-2* 3'-end |
| S11 | 76 | 103 | 133-234 | 61738-61639 | *AhHMA4-1*$_P$ |
| S12 | 77 | 71 | 410-480 | 167443-167509 | intergenic region At2g19120-2(c)/*AhHMA4-3* |

[1]Sequence identities and hit lengths are given for the top-scoring non-self hits running amplicon sequences (predicted based on BAC contig) as queries against the *HMA4* BAC contig in BLASTN with default settings at NCBI (Genbank accession numbers EU382073.1 and EU382072.1; contig length 289,768 bp [8]).

[2]All three genes (At2g19010/S2, At2g19050 and At2g19060) encode putative GDSL-motif lipase/hydrolase family proteins.

Note that sequences corresponding to S1 and S13 are not present in the *HMA4* BAC contig. Red fonts: segments comprising repeated sequence stretches present in several, almost identical copies in the genomic *HMA4* region. r.c.: reverse complement. -: no significant hit.

**B**. Sequence similarities between amplified segments of *A. halleri* and sequences in the *A. thaliana* and *A. lyrata* ssp. *lyrata* genomes.

| **Top hits** | *A. thaliana* | | | *A. lyrata* | | |
|---|---|---|---|---|---|---|
| Loci | Sequence identity (%)[1] | Hit length (nt)[1] | Annotation | Sequence identity (%)[1] | Hit length (nt)[1] | Annotation |
| S1 | 92 | 328 | At2g19750 | 96 | 149 | GENE ID: 9322332 ARALYDRAFT_900373 |
| S2 | 96 | 217 | At2g19010 | 99 | 217 | GENE ID: 9305466 ARALYDRAFT_491739 |
| S3 | 92 | 546 | At2g19090 | 94 | 540 | GENE ID: 9322308 ARALYDRAFT_480823 |
| S4 | 91 | 64 | 5'-UTR of *AtHMA4* | 97 | 33 | GENE ID: 9322307 ARALYDRAFT_480822[2] |
| S5 | 92 | 690 | *AtHMA4* | 95 | 692 | GENE ID: 9322307 ARALYDRAFT_480822[2] |
| | | | | 95 | 692 | GENE ID: 9330132 ARALYDRAFT_473917[3] |
| S6 | 79 | 186 | BAC t20k24 (in *AtHMA4* promoter) | 85 | 197 | *Arabidopsis lyrata* clone JGIFAFI-61E14 |
| S7 | 92 | 690 | *AtHMA4* | 95 | 692 | GENE ID: 9322307 ARALYDRAFT_480822[2] |
| | | | | 95 | 692 | GENE ID: 9330132 ARALYDRAFT_473917[3] |
| S8 | 96 | 632 | At2g19120 | - | - | - |
| S9 | 71 | 295 | BAC t20k24 (in *AtHMA4* promoter) | 82 | 95 | GENE ID: 9307927 ARALYDRAFT_909928 |
| S10 | 92 | 690 | *AtHMA4* | 95 | 692 | GENE ID: 9322307 ARALYDRAFT_480822[2] |
| | | | | 95 | 692 | GENE ID: 9330132 ARALYDRAFT_473917[3] |
| S11 | 92 | 235 | At2g19150 | 98 | 236 | GENE ID: 9322304 ARALYDRAFT_480819 |
| S12 | 92 | 450 | At2g19160 | 90 | 454 | GENE ID: 9320242 ARALYDRAFT_480818 |
| S13 | 96 | 222 | At2g19490 | 83 | 289 | GENE ID: 9320055 ARALYDRAFT_480524 |
| **Second hits** | *A. thaliana* | | | *A. lyrata* | | |
| Loci | Sequence identity (%)[1] | Hit length (nt)[1] | Annotation | Sequence identity (%)[1] | Hit length (nt)[1] | Annotation |
| S1 | 89 | 162 | At2g19740 | 75 | 226 | Flowering locus T promoter (FN813300.1) |
| S2 | 72 | 186 | At2g19060 | 71 | 211 | GDSL-motif lipase/hydrolase family protein |
| | < 80 | variable | multiple hits[4] | | | |
| S3 | 84 | 224 | At4g30130 (segmental duplication) | 73 | 102 | GENE ID: 9305000 ARALYDRAFT_656131 |
| S4 | - | - | - | - | - | - |
| S5 | 70 | 207 | At4g30110 - *AtHMA2* (segmental duplication) | 70 | 207 | GENE ID: 9303439 ARALYDRAFT_657042 |
| S6 | - | - | - | - | - | - |
| S7 | | 207 | At4g30110 - *AtHMA2* (segmental duplication) | 70 | 207 | GENE ID: 9303439 ARALYDRAFT_657042 |
| S8 | 86 | 556 | At4g30100 (segmental duplication) | - | - | - |
| S9 | - | - | - | - | - | - |
| S10 | | 207 | At4g30110 - *AtHMA2* (segmental duplication) | 70 | 207 | GENE ID: 9303439 ARALYDRAFT_657042 |
| S11 | 76 | 158 | At2g47280 | - | - | - |
| S12 | 77 | 339 | At4g30060 (segmental duplication) | 76 | 366 | GENE ID: 9305472 ARALYDRAFT_491750 |
| S13 | 86 | 327 | At2g19480 | 88 | 41 | GENE ID: 9328930 ARALYDRAFT_471744 |

[1]Sequence identities and hit lengths are given for the two top-scoring hits from BLASTN with default settings using amplicon sequences as queries against all *A. thaliana* and *A. lyrata* ssp. *lyrata* sequences at NCBI.

[2]*AlHMA4*.

[3]Truncated pseudogene copy of *HMA4* present only in *A. lyrata* and corresponding to the 3'-end of *HMA4* (see Figure S1).

[4]Part of the At2g19010/At19020 intergenic region of the *A. halleri* S2 sequences contains repeats found at multiple sites on multiple chromosomes of *A. thaliana*.

Red fonts: segments comprising repeated sequence stretches present in several, almost identical copies in the *HMA4* genomic region of *A. halleri*. r.c.: reverse complement.-: no significant hit.
