# Supplementary material for: Hard Selective Sweep and Ectopic Gene Conversion in a Gene Cluster Affording Environmental Adaptation
Source: PLoS Genet. 2013 Aug 22;9(8):e1003707. doi: 10.1371/journal.pgen.1003707 (PMC3749932; doi:10.1371/journal.pgen.1003707)
Supplement: Table S4 — Robustness of π estimation. (PDF) [file pgen.1003707.s011.pdf]

Table S4. Robustness of π estimation.

A. Estimation of error in π resulting from uncertainties in the assignment of sequences to *HMA4* gene copy (for S5, S7 and S10).

	Genotypes								
	All *A. halleri* ssp. *halleri*			Harz Mountains			Thuringian Forest		
Locus	*N*	No. of sites	π	*N*	No. of sites	π	*N*	No. of sites	π
S5 only (*HMA4-1*)	35	1320	0.0042	22	1320	0.0042	11	1336	0.0025
S5 + ambiguous S5/S10	40	1320	0.0043	25	1320	0.0042	13	1336	0.0033
S7 anchored based on S8	34	1327	0.0032	22	1327	0.0032	10	1327	0.0020
S7 only (*HMA4-2*)	37	1327	0.0031	23	1327	0.0033	12	1327	0.0019
S7 + ambiguous S7/S10	39	1327	0.0032	25	1327	0.0033	12	1327	0.0019
S10 only (*HMA4-3*)	34	1336	0.0035	21	1336	0.0039	11	1349	0.0035
S10 + ambiguous S5/S10	41	1320	0.0036	26	1320	0.0039	13	1349	0.0036

No. of sites are numbers of informative sites, excluding those with gaps. Sequences that could not be assigned to a single *HMA4* gene copy are named ambiguous (see Figure 4, Figure S4).

B. Dependence of π on variation in sampling.

	Segment												
Genotypes	S1	S2	S3	S4	S5	S6	S7	S8	S9	S10	S11	S12	S13
All *A. halleri* ssp. *halleri*	0.0049	0.0059	0.0021	0.0018	0.0042	0.0010	0.0032	0.0052	0.0001	0.0035	0.0008	0.0069	0.0091
Harz Mountains	0.0033	0.0063	0.0026	0.0023	0.0042	0.0006	0.0032	0.0034	0.0001	0.0039	0.0007	0.0068	0.0098
Thuringian Forest	0.0012	0.0066	0.0004	0.0003	0.0025	0.0011	0.0020	0.0062	0.0001	0.0035	0.0005	0.0057	0.0061

Grey shade: amplicons in triplicated region (*HMA4* and two downstream genes, see Figure 1). Red fonts: segments comprising repeated sequence stretches present in several, almost identical copies in the *HMA4* genomic region.
