# Supplementary material for: Hard Selective Sweep and Ectopic Gene Conversion in a Gene Cluster Affording Environmental Adaptation
Source: PLoS Genet. 2013 Aug 22;9(8):e1003707. doi: 10.1371/journal.pgen.1003707 (PMC3749932; doi:10.1371/journal.pgen.1003707)
Supplement: Table S5 — Analyses of Molecular variance (AMOVA) in A. halleri. (PDF) [file pgen.1003707.s012.pdf]

Table S5. Analyses of Molecular Variance (AMOVA) in *A. halleri*.

A. Collection sites were assigned to three geographic regions (referred to as groups): Harz Mountains (*A. halleri* ssp. *halleri*: 1 to 5; see Table 1), Thuringian Forest (*A. halleri* ssp. *halleri*: 6, 7), and Japan (*A. halleri* ssp. *gemmifera*: 9).

Segment	Source of variation	*df*	Sum of squares	Variance components	Percentage of variation	Fixation Indices	*P* value
S1	Between groups	1	44.8	3.76	57.1	F_{CT}: 0.57	0.1515 (± 0.0137)
	Between sites within groups	4	29.1	1.21	18.4	F_{SC}: 0.43	0.0049 (± 0.0020)
	Within sites	24	38.8	1.62	24.5	F_{ST}: 0.75	$< 5 \cdot 10^{-6}$ ($\pm < 5 \cdot 10^{-6}$)
	Total	29	112.8	6.59			
S2	Between groups	2	26.0	0.77	22.0	F_{CT}: 0.22	0.1652 (± 0.0126)
	Between sites within groups	5	33.2	0.99	28.3	F_{SC}: 0.36	0.0059 (± 0.0022)
	Within sites	30	52.2	1.74	49.7	F_{ST}: 0.50	$< 5 \cdot 10^{-6}$ ($\pm < 5 \cdot 10^{-6}$)
	Total	37	111.3	3.50			
S3	Between groups	2	10.3	0.35	21.4	F_{CT}: 0.21	0.0254 (± 0.0040)
	Between sites within groups	5	10.2	0.19	11.7	F_{SC}: 0.15	0.1241 (± 0.0128)
	Within sites	30	32.7	1.09	66.8	F_{ST}: 0.33	$< 5 \cdot 10^{-6}$ ($\pm < 5 \cdot 10^{-6}$)
	Total	37	53.2	1.63			
S4	Between groups	2	17.4	0.69	30.6	F_{CT}: 0.31	0.0078 (± 0.0031)
	Between sites within groups	5	11.9	0.21	9.4	F_{SC}: 0.14	0.2033 (± 0.0153)
	Within sites	30	40.2	1.34	59.9	F_{ST}: 0.40	0.0010 (± 0.0010)
	Total	37	69.6	2.24			
S6[a]	Between groups	1	2.4	0.12	25.2	F_{CT}: 0.25	0.1134 (± 0.0098)
	Between sites within groups	5	2. 7	0.05	10.3	F_{SC}: 0.14	0.0635 (± 0.0065)
	Within sites	29	8. 7	0.30	64.5	F_{ST}: 0.35	0.0020 (± 0.0014)
	Total	35	13. 8	0.46			
S9[a]	Between groups	1	0.1	0.00	0.8	F_{CT}: 0.01	0.6188 (± 0.0151)
	Between sites within groups	5	0.5	-0.07	-5.1	F_{SC}: -0.05	0.6901 (± 0.0084)
	Within sites	25	3.2	0.13	104.2	F_{ST}: -0.04	0.6843 (± 0.0154)
	Total	31	3.9	0.12			
S11	Between groups	2	8.8	0.44	44.6	F_{CT}: 0.45	0.0049 (± 0.0020)
	Between sites within groups	5	3.5	0.04	4.2	F_{SC}: 0.08	0.0841 (± 0.0082)
	Within sites	28	14.1	0.50	51.2	F_{ST}: 0.49	$< 5 \cdot 10^{-6}$ ($\pm < 5 \cdot 10^{-6}$)
	Total	35	26.4	0.98			
S12	Between groups	2	45.0	1.23	15.3	F_{CT}: 0.14	0.0860 (± 0.0077)
	Between sites within groups	5	60.2	1.33	16.5	F_{SC}: 0.20	0.0293 (± 0.0048)
	Within sites	30	164.7	5.49	68.2	F_{ST}: 0.31	$< 5 \cdot 10^{-6}$ ($\pm < 5 \cdot 10^{-6}$)
	Total	37	270.0	8.06			
S13	Between groups	2	40.8	0.74	8.6	F_{CT}: 0.09	0.2248 (± 0.0104)
	Between sites within groups	5	81.9	2.32	27.2	F_{SC}: 0.30	0.0078 (± 0.0034)
	Within sites	28	153.2	5.47	64.1	F_{ST}: 0.36	$< 5 \cdot 10^{-6}$ ($\pm < 5 \cdot 10^{-6}$)
	Total	35	275.9	8.53			

B. Collection sites were assigned to four geographic regions (referred to as groups): Groups were as in (**a**) above, and additionally including France as a fourth region (group) represented by Auby (*A. halleri* ssp. *halleri*: 8; see Table 1).

Segment	Source of variation	*df*	Sum of squares	Variance components	Percentage of variation	Fixation Indices	*P* value
S1	Between groups	n.a.	n.a.	n.a.	n.a.	n.a.	n.a.
	Between sites within groups	n.a.	n.a.	n.a.	n.a.	n.a.	n.a.
	Within sites	n.a.	n.a.	n.a.	n.a.	n.a.	n.a.
	Total	n.a.	n.a.	n.a.	n.a.	n.a.	n.a.
S2	Between groups	3	26.9	0.53	16.6	F_{CT}: 0.17	0.2493 (± 0.0132)
	Between sites within groups	5	33.2	1.00	31.5	F_{SC}: 0.37	0.0059 (± 0.0022)
	Within sites	31	52.2	1.68	52.3	F_{ST}: 0.48	0.0010 (± 0.0010)
	Total	39	112.2	3.22			
S3	Between groups	3	10.8	0.25	16.5	F_{CT}: 0.16	0.0616 (± 0.0072)
	Between sites within groups	5	10.2	0.19	11.9	F_{SC}: 0.14	0.1241 (± 0.0117)
	Within sites	31	34. 7	1.12	71.5	F_{ST}: 0.28	0.0039 (± 0.0018)
	Total	39	55.6	1.56			
S4	Between groups	3	18.4	0.56	26.3	F_{CT}: 0.27	0.0371 (± 0.0057)
	Between sites within groups	5	11.9	0.21	10.3	F_{SC}: 0.14	0.2014 (± 0.0125)
	Within sites	31	41.2	1.33	63.4	F_{ST}: 0.37	0.0078 (± 0.0034)
	Total	39	71.6	2.11			
S6[a]	Between groups	3	50.0	1.99	81.3	F_{CT}: 0.81	0.0371 (± 0.0048)
	Between sites within groups	5	2.7	0.02	0.8	F_{SC}: 0.04	0.0743 (± 0.0069)
	Within sites	33	14. 7	0.44	17.9	F_{ST}: 0.82	$< 5 \cdot 10^{-6}$ ($\pm < 5 \cdot 10^{-6}$)
	Total	41	67.1	2.44			
S9[a]	Between groups	3	84.0	3.77	82.9	F_{CT}: 0.83	0.0987 (± 0.0110)
	Between sites within groups	5	0.5	-0.20	-4.5	F_{SC}: -0.26	0.7077 (± 0.0157)
	Within sites	29	28.5	0.98	21.6	F_{ST}: 0.78	$< 5 \cdot 10^{-6}$ ($\pm < 5 \cdot 10^{-6}$)
	Total	37	113.0	4.55			
S11	Between groups	n.a.	n.a.	n.a.	n.a.	n.a.	n.a.
	Between sites within groups	n.a.	n.a.	n.a.	n.a.	n.a.	n.a.
	Within sites	n.a.	n.a.	n.a.	n.a.	n.a.	n.a.
	Total	n.a.	n.a.	n.a.	n.a.	n.a.	n.a.
S12	Between groups	3	59.8	1.37	16.8	F_{CT}: 0.17	0.0753 (± 0.0081)
	Between sites within groups	5	60.2	1.34	16.5	F_{SC}: 0.20	0.0283 (± 0.0059)
	Within sites	31	168.2	5.42	66.7	F_{ST}: 0.33	$< 5 \cdot 10^{-6}$ ($\pm < 5 \cdot 10^{-6}$)
	Total	39	288.3	8.14			
S13	Between groups	3	50.6	0.60	7.3	F_{CT}: 0.07	0.2698 (± 0.0169)
	Between sites within groups	5	81.9	2.36	28.6	F_{SC}: 0.31	0.0039 (± 0.0018)
	Within sites	29	153.2	5.28	64.0	F_{ST}: 0.36	$< 5 \cdot 10^{-6}$ ($\pm < 5 \cdot 10^{-6}$)
	Total	37	285.7	8.25			

[a]excluding *A. halleri* ssp. *gemmifera*. n.a.: not applicable (data for S1 and S11 are missing for the individual from Auby; see Table S1)
