# Supplementary material for: Hard Selective Sweep and Ectopic Gene Conversion in a Gene Cluster Affording Environmental Adaptation
Source: PLoS Genet. 2013 Aug 22;9(8):e1003707. doi: 10.1371/journal.pgen.1003707 (PMC3749932; doi:10.1371/journal.pgen.1003707)
Supplement: Table S6 — Sequences and reaction efficiencies of primer pairs used for quantitative PCR determination of genomic copy number. (PDF) [file pgen.1003707.s013.pdf]

Table S6. Sequences and reaction efficiencies of primer pairs used for quantitative PCR determination of genomic copy number.

Amplicon		Species: *A. halleri*	*A. lyrata* ssp. *lyrata*	*A. thaliana* (Col-0)	Primer efficiency†
HMA4	Forward	GCTGCAGCGATGAAAAACAAAC	GCTGCAACGATGAAAAGCAAAC	GCTGCAGCGATGAAAAACAAAC	1.91 ± 0.01<br>*n* = 290
	Reverse	TCCATACAACATCCCGAGGAAC	TCCATACAACATCCCGAGGAAC	TTCACACAACATCCCGAGGAGC	
FRD3 5'	Forward	TGGCAGAGGAAGACACGATG	TGGCAGAGGAAGACACGATG	TGGCAGAGGAAGACACGATG	1.91 ± 0.01<br>*n* = 276
	Reverse	TGGCTTTGTTCGCTTCTTCTTT	TGGCTTTGTTCGCTTCTTCTTT	TGGCTTTGTTTGCTTCTTCTTT	
FRD3 3'	Forward	TTTATAGCAGCCACGCAGCC	TTTATAGCAGCCACGCAACC	TTTATAGCAGCAACGCAGCC	1.89 ± 0.01<br>*n* = 294
	Reverse	TCCATCCAATACAAAGGCGAGT	TCCATCCAATACAAAGGCGAGT	TCCATCCAATACAAAGGCGAGA	
S13	Forward	ACTTCTGGGTAGTTGGAATTTTCC	ACTTCTGGGTAGTTGGAATTTTCC	ACTTCTGGGTAGTTGGAATTTTCC	1.89 ± 0.01<br>*n* = 276
	Reverse	CGTTTCGGAGAATCCTCGC	CGTTTCGGAGAATCCTCGC	CGTTTCGGAGAATCCTCGC	

All primer sequences are given in 5'- to 3'-direction. The *HMA4* and S13 primers for *A. halleri* were designed based on an alignment of all S5/S7/S10 and S13 consensus sequences (see Figure 1, Table S1), respectively, in order to ensure the absence of polymorphisms within primer binding sites. The *FRD3* primers for *A. halleri* were designed based on available sequences from the Lan3.1 individual of the Langelsheim population [12]. The *A. thaliana* and *A. lyrata* ssp. *lyrata* primers were designed based on available genome sequences [39,73].

†: arithmetic mean ± s. e. m., *n* equals the number of replicate PCR reactions per amplicon.
